# The anti-cancer agent APR-246 can activate several programmed cell death processes to kill malignant cells

**DOI:** 10.1038/s41418-023-01122-3

**Published:** 2023-02-04

**Authors:** Zilu Wang, Huimin Hu, Luuk Heitink, Kelly Rogers, Yue You, Tao Tan, Connie Li Wai Suen, Alex Garnham, Hao Chen, Elizabeth Lieschke, Sarah T. Diepstraten, Catherine Chang, Tianwei Chen, Diane Moujalled, Kate Sutherland, Guillaume Lessene, Oliver M. Sieber, Jane Visvader, Gemma L. Kelly, Andreas Strasser

**Affiliations:** 1 The Walter and Eliza Hall Institute of Medical Research, Melbourne, VIC 3052 Australia; 2 Department of Medical Biology, University of Melbourne, Melbourne, VIC 3052 Australia; 3 Department of pharmacology and Therapeutics, University of Melbourne, Melbourne, VIC 3052 Australia

**Keywords:** Cancer, Cell biology, Biochemistry

## Abstract

Mutant TP53 proteins are thought to drive the development and sustained expansion of cancers at least in part through the loss of the wild-type (wt) TP53 tumour suppressive functions. Therefore, compounds that can restore wt TP53 functions in mutant TP53 proteins are expected to inhibit the expansion of tumours expressing mutant TP53. APR-246 has been reported to exert such effects in malignant cells and is currently undergoing clinical trials in several cancer types. However, there is evidence that APR-246 may also kill malignant cells that do not express mutant TP53. To support the clinical development of APR-246 it is important to understand its mechanism(s) of action. By establishing isogenic background tumour cell lines with different TP53/TRP53 states, we found that APR-246 can kill malignant cells irrespective of their TP53/TRP53 status. Accordingly, RNAseq analysis revealed that treatment with APR-246 induces expression of the same gene set in *Eμ-Myc* mouse lymphoma cells of all four possible TRP53 states, wt, wt alongside mutant, knockout and knockout alongside mutant. We found that depending on the type of cancer cell and the concentration of APR-246 used, this compound can kill malignant cells through induction of various programmed cell death pathways, including apoptosis, necroptosis and ferroptosis. The sensitivity of non-transformed cells to APR-246 also depended on the cell type. These findings reveal that the clinical testing of APR-246 should not be limited to cancers expressing mutant TP53 but expanded to cancers that express wt TP53 or are TP53-deficient.

## Introduction

TP53 (called TRP53 in mouse) is a tumour suppressor that is encoded by the most frequently mutated gene in human cancer, with ~50% of sporadic human cancers carrying mutations in *TP53* [1]. Moreover, germline mutations in the *TP53* gene, the cause of a syndrome called Li-Fraumeni, predispose carriers to tumour development early in life [2]. As a transcription factor, TP53/TRP53 is able to transactivate ~500 target genes directly, some of which encode known regulators of diverse cellular responses, such as cell cycle arrest, cellular senescence and apoptotic cell death [3–5]. Mutations in *TP53* are reported to drive the development and sustained expansion of tumours through three different, albeit not mutually exclusive properties: loss of wild-type (wt) TP53 function (LOF), dominant-negative effects (DNE) on wt TP53 (which are seen during the early stages of neoplastic transformation when cells express both mutant TP53/TRP53 and wt TP53/TRP53) and neomorphic gain-of-function (GOF) effects. The importance of the LOF, i.e. the inability of mutant TP53/TRP53 proteins to transactivate the target genes that are regulated by wt TP53/TRP53, in tumorigenesis has been established by the demonstration that *Trp53* gene-deficient mice develop tumours at an early age [6]. Moreover, studies with genetically engineered mice have shown that restoring wt TRP53 function in cancers that were caused by its absence potently inhibits tumour expansion by inducing either apoptosis or cell cycle arrest/cellular senescence, dependent on the tumour type [7–9]. It has therefore been postulated that drugs that can restore wt TP53 transcriptional activities in human tumour cells expressing mutant TP53 would cause cell proliferation arrest, cellular senescence and/or apoptotic cell death with consequent therapeutic benefit. APR-246 was reported to exert such an activity, and this compound is currently undergoing clinical trials in several cancer types but only in cases expressing mutant TP53 (ClinicalTrials.gov Identifier: NCT02999893, NCT03931291, NCT04214860 and NCT04383938) [10].

APR-246 is reported to work by its conversion in cells into the reactive electrophile methylene quinuclidinone (MQ). MQ reacts with cysteine residues C124, C229 and C277 in the core of mutant TP53 proteins and is reported to thereby change their conformation from “mutant” to “wt”, resulting in the restoration of transactivation of wt TP53 target genes that inhibit tumour growth [11, 12]. Notably, in addition to mutant TRP53, MQ can modify a very large number of proteins in cells, including many enzymes. This is likely to impact a broad range of cellular processes, as for example shown for redox homeostasis in cancer cells through inhibition of thioredoxin reductase, thioredoxin, glutaredoxin, ribonucleotide reductase and depletion of glutathione [13–16]. Accordingly, there is also evidence that APR-246 can kill tumour cells independent of mutant TP53. For example, it was reported that APR-246 can induce apoptosis in colorectal cancer cells irrespective of the TP53 state [17] or to directly activate wt TP53 in melanoma [18]. Many of these previous studies compared different cancer cells with different TP53/TRP53 states rather than isogenic cancer cells with different TP53/TRP53 states. This makes it difficult to delineate the role of mutant TP53/TRP53 in APR-246 induced killing. Here we compare for the first time the response of isogenic cancer cells with different TP53/TRP53 states to APR-246. This revealed that this drug can kill cancer cells independently of mutant TP53/TRP53. Importantly, we discovered that APR-246 can kill malignant cells by inducing different cell death pathways. We also for the first time examine the effect of APR-246 on non-malignant cells using primary murine and human cells and organoids. Collectively, our findings provide valuable insight for improving the efficacy and safety of APR-246 that will help advance the clinical development of this compound or related agents.

## Materials and methods

### Reagents

APR-246 was provided by Aprea (Stockholm, Sweden) and dissolved in DMSO to prepare the stock. Nutlin-3a was purchased from Selleckchem and dissolved in DMSO. The BH3-mimetic compounds ABT-199 (inhibitor of BCL-2), ABT-737 (inhibitor of BCL-2, BCL-XL and BCL-W) and S63845 (inhibitor of MCL-1) were purchased from Active Biochem and stock was prepared as previously described [19]. TSI compounds (TNFα, the SMAC mimetic compound A and the broad-spectrum caspase inhibitor IDN-6556) were provided by Prof. Guillaume Lessene (WEHI) and prepared as previously described [20]. The inducer of ferroptosis, RSL-3, and the inhibitor of ferroptosis, ferrostatin-1, were purchased from Sigma-Aldrich and dissolved in DMSO.

### Cell culture

*Eμ-Myc* mouse lymphoma cells were cultured in the high-glucose version of Dulbecco’s modified Eagle’s medium (DMEM, Gibco) supplemented with 10% foetal calf serum (FCS, Sigma-Aldrich), 100 mM L-asparagine (Sigma-Aldrich), 50 μM β-mercaptoethanol (β-ME, Sigma-Aldrich) and 100 U/mL Penicillin-Streptomycin (Pen/Strep) (Gibco). MDA-MB-231, SW620 and HT29 cells were maintained in DMEM supplemented with 10% FCS and 100 U/mL Pen/Strep. U937 cells were cultured in RPMI 1640 medium (Gibco) supplemented with 10% FCS and 100 U/mL Penicillin-Streptomycin. Rael-BL cells were maintained in RPMI 1640 medium supplemented with 10% FCS, 2 mM L-glutamine, 1 mM sodium pyruvate (Gibco), 50 μM α-thioglycerol and 100 U/mL Pen/Strep. BEAS-2B cells were cultured with BEGM bronchial epithelial cell growth medium Bulletkit (Lonza). Mouse B cells were isolated from spleen cell suspensions and activated for 48 h in RPMI 1640 medium supplemented with 10% FCS, 1× Glutamax (Gibco), 25 mM HEPES (Gibco), non-essential amino acids (Gibco), 1 mM sodium pyruvate, 50 μM β-ME, Pen/Strep, 10 μg/mL anti-mouse CD40 antibody (BD Biosciences), 100 U/mL IL-4 (BD Biosciences) and 15 μg/mL anti-mouse IgM Fab_2_ antibody fragments (Jackson ImmunoResearch). Contamination of cell cultures with mycoplasma was monitored routinely. Cell lines had been authenticated by STR DNA profiling analysis before use in the experiments presented here.

### Mouse mammary gland derived organoids

All experiments with mice were conducted under approval of and following the guidelines of the Walter and Eliza Hall Institute’s Animal Research Ethics Committee. Mammary glands from C57BL/6 female mice were dissected and processed as previously described [21]. Mammary gland organoids were established from isolated basal cells as previously described [22]. FACS sorted CD24^+^/CD29^+^ basal cells were resuspended in ice-cold BME medium (R&D Research) and maintained in advanced DMEM/F12 medium (Gibco) supplemented with Pen/Strep, 1× Glutamax, 10 mM HEPES, 5 μg/mL Insulin (Roche), 100 ng/mL Hydrocortisone (Sigma Aldrich), 1× B27 (ThermoFisher), 1.25 µM N-Acetylcysteine (Sigma Aldrich), 50 ng/mL EGF (Sigma Aldrich), 5 ng/mL FGF-basic (R&D), 10 ng/mL FGF10 (Peprotech), 4 µg/mL Heparin (SigmaAldrich) and R-Spondin2 conditioned medium (0.5%, generated in-house). Medium was refreshed every 48 h. After 6 days, organoids were disrupted into single cell suspensions using TrypLE express (Thermo Fisher) and forced pipetting. Single cells were plated on a 96-well plate and cultured for 48 h before exposure to various concentrations of APR-246 for the next 48 h. Organoid cell viability was assessed using Cell Titre Glow 3D (Promega).

### Human colon derived organoids

Human intestinal tissues were collected, and cells were isolated as previously described [23]. Cells were embedded in Matrigel (Corning) and maintained in human IntestiCult organoid growth medium (Stemcell) containing 10 μM Y-27632. Single cells were plated into a 96-well plate and cultured for 72 h before being exposed to the indicated concentrations of APR-246 for the next 48 h. Organoid cell viability was assessed by using Cell Titre Glow 3D. This study was conducted in accordance with the Declaration of Helsinki, the NHMRC Statement on Ethical Conduct in Human Research, and Institutional Human Research Ethics approval (HREC 2016.249, Walter and Eliza Hall Institute of Medical Research).

### Virus packaging and cell infection

Genes of interest were deleted/mutated by using our CRISPR/Cas9 gene editing platform [24]. Virus packaging was performed as previously described [24]. The sequences of the sgRNAs used in this study are as follows:

human *TP53*: 5ʹ - GAGCGCTGCTCAGATAGCGA

mouse *Trp53*: 5ʹ - GGCAACTATGGCTTCCACCT

mouse *Puma*: 5ʹ - ACTCTAAGTGCTGCTGGGCTGG

mouse *Noxa*: 5ʹ - GTTGAGCTGCGAACTCAGGTGG

mouse *Bim*: 5ʹ - GACAATTGCAGCCTGCTGAG

mouse *Bax*: 5ʹ - CAGTTCATCTCCAATTCGC

mouse *Bak*: 5ʹ - TCCATCTCGGGGTTGGCAG

human *BAX*: 5ʹ- CTGCAGGATGATTGCCGCCG

human *BAK*: 5ʹ – GGCCATGCTGGTAGACGTGT

mouse *Ninj1*: 5ʹ – ACCACAAGGGGCACGAAGAAGG

human *NINJ1*: 5ʹ – ACCGAGGAGTACGAGCTCAA

human *MLKL*: 5ʹ- TCCCGGAGCTCTCGCTGTTACTTC

mouse *Mlkl*: 5ʹ- GCGTCTAGGAAACCGTGTGC

To achieve the infection of cells with viruses containing specific sgRNAs, 5 × 10^5^ adherent cells were seeded into a well of a 6 well plate and incubated with 1 mL virus supernatant overnight. For suspension cells, 1 × 10^5^ cells were mixed with 3 mL virus supernatant and spun at 2200 rpm for 2 h. After 72 h, the efficiency of deletion of the gene of interest was checked by Western blotting and DNA sequence analysis.

### Western blotting

Protein extracts were collected by lysing cells in RIPA buffer (150 mM sodium chloride, 50 mM pH 8.0 Tris-HCl, 1% NP-40, 0.5% sodium deoxycholate, 0.1% SDS). A total of 20 μg of proteins were loaded into the well of a NuPage protein gel (ThermoFisher) and proteins were separated based on their molecular weight by electrophoresis in MES-SDS running buffer (SigmaAldrich). Proteins were then transferred onto a nitrocellulose membrane by using the iBlot stacks system (Thermo Fisher). After blocking non-specific antibody binding sites on the membranes with PBS containing 5% skim milk and 0.05% Tween 20 (SigmaAldrich), the membranes were incubated with primary antibody, followed by three washing steps in PBS with 0.05% Tween 20. The membranes were then incubated with secondary antibody conjugated to horseradish peroxidase (HRP). After a further three washes the bands were visualised by applying the Immobilon Forte Western HRP substrate (Sigma-Aldrich), using Chemidoc imaging system (Bio-Rad). The primary antibodies used in this study included ones against human TP53 (DO-1, Santa Cruz), mouse TRP53 (CM5, Leica), mouse PUMA (ProSci), mouse BIM (Enzo), mouse NINJ1 (ThermoFisher), mouse BAX (5B7, Sigma-Aldrich), mouse BAK (TC102, Merck) and β-ACTIN (AC40, Sigma-Aldrich), the latter used as a loading control. Antibodies against mouse and human MLKL were raised in-house [25]. The secondary antibodies used in this study included HRP conjugated goat anti-mouse IgG, HRP conjugated goat anti-rabbit IgG and HRP conjugated goat anti-rat IgG (all from Southern Biotech).

### RNA sequencing

Total RNA was extracted by using Trizol (ThermoFisher) from *Eμ-Myc* mouse lymphoma cells with four different TRP53 states either treated with APR-246 or vehicle. The mRNA libraries were prepared by using the TruSeq RNA library prep kit 2 (illumina) and then sequenced on a NextSeq 500 instrument using High Output kit (150 cycles; Illumina).

For all data sets sequencing reads were aligned to the mouse reference genome using STAR package v2.5.3 [26]. After alignment, successfully mapped reads were summarised into gene-level counts using FeatureCounts [27]. For all samples, an average of 70% of read pairs were assigned to a gene. Differential gene expression analyses were performed using the limma v3.46.0 [28] and edgeR v3.32.1 [29] software packages. Lowly expressed genes were filtered out using the filterByExpr function in edgeR, which resulted in 10,902 genes being retained for the following analysis. Compositional differences between each sample were normalised using the trimmed mean of M-values (TMM) method [30]. Read counts were converted to log_2_-counts-per-million (logCPM) and differential expression of genes between cells of each TRP53 state treated or not treated with APR-246 was assessed using linear models and robust empirical Bayes moderated t-statistics with a trended prior variance relative to a fold-change threshold of 1.2 (limma-trend pipeline with TREAT) [31]. Mean difference plots were generated using the ggplots (v2.2.1) and expression plots were generated using Glimma (v1.8.2) [32].

### Cell viability assays

In total, 3 × 10^4^ suspension cells or 1 × 10^4^ adherent cells were seeded into a well of a flat-bottom 96 well plate. Cells were treated for 48 h with the indicated concentrations of APR-246 for 48 h or for 24 h with 10 μM nutlin-3a. Cells were then stained with propidium iodide (PI). The dead cells (PI positive) were measured by flow cytometry and data were analysed by using the FlowJo software (BD Life Sciences).

For organoids, 5×10^3^ cells were mixed with the same volume of Celltiter Glo 3D and shaken for 15 min at room temperature. The florescence of each sample was measured by using a microplate reader CLARIOstar Plus (BMG Labtech).

### DAPI and Annexin V staining

3×10^4^ cells were seeded into a flat-bottom 96 well plate and treated with 15 μM of APR-246 for 24 h. Cells were harvested and stained with DAPI (1 μg/mL) and Alexa Fluor 647-conjugated Annexin V (made in house) for 15 min on ice. The cells were then analysed using an LSR IIW (BD) flow cytometer. Data were examined using FlowJo v10 software.

### Live cell imaging

In total, 1 × 10^5^ cells were seeded into an m-slide 8 well plate (iBidi) and treated with the indicated concentrations of APR-246. Cells were immediately stained with PI and APC conjugated Annexin V (produced in-house). Images were captured every 5 or 10 min for 12 h using a laser scanning confocal microscope LSM 980 Airyscan (Zeiss). Data were analysed by using Imaris and Image J software.

### LDH release assay

The release of LDH was measured by using the CytoTox Non-Radioactive Cytotoxicity assay (Promega). In brief, 3 × 10^4^ cells were seeded into the well of a flat-bottom 96 well plate and treated with the specified doses of APR-246 for 24 h or 48 h, respectively. In total, 50 μL supernatant was mixed with the same volume of CytoTox Reagent and then incubated at room temperature for 30 min (avoiding light). After adding 50 μL of Stop solution, the absorbance of each well was measured at 492 nm by using a CLARIOstar Plus plate reader (BMG Labtech).

### Sequencing of *Trp53* exons by MiSeq

Genomic DNA from cancer cell lines was purified using the DNeasy Blood and Tissue Kit (Qiagen). Each exon of mouse *Trp53* was amplified by PCR. Primers are listed in Supplementary Table S2. A second indexing PCR was performed to barcode samples for Illumina sequencing. The PCR amplicons were pooled, and purified using AMPure XP beads (Beckman Coulter), followed by sequencing on a MiSeq instrument (Illumina).

### Statistical analysis

GraphPad Prism was used for statistical analysis. Error bars indicate the standard deviation of results from 3 or more independent experiments. Two-tailed *t* tests were used to compare between two data sets.

## Results

### APR-246 can kill *Eμ-Myc* mouse lymphoma cells regardless of their TRP53 state

**Fig. 1:**
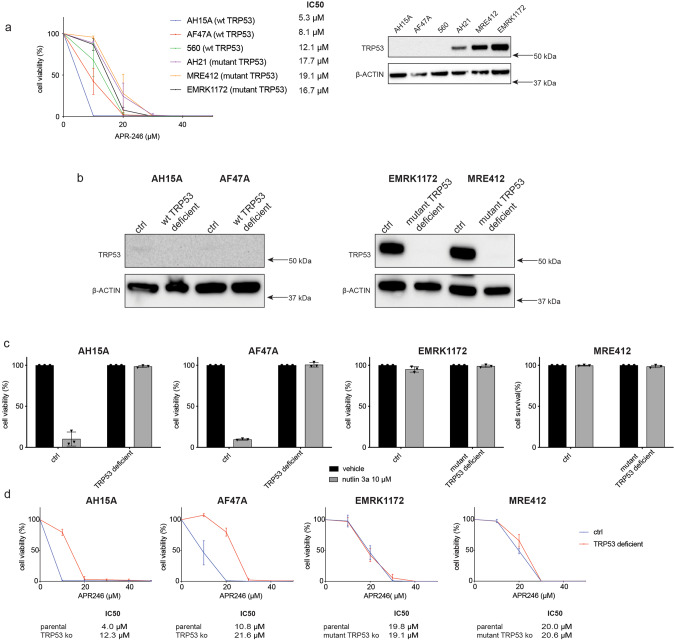
APR-246 can kill *Eμ-Myc* mouse lymphoma cells irrespective of their TRP53 status. **a** Testing the responses of a panel of *Eμ-Myc* mouse lymphoma cell lines to the indicated concentrations of APR-246. Western blot analysis showing lymphoma cells expressing wt TRP53 or mutant TRP53. Note wt TRP53 is expressed at very low levels whereas the mutant TRP53 protein is stabilised. Probing for β-ACTIN was used as a loading control. The indicated lymphoma cells were treated for 48 h with the indicated concentrations of APR-246 and cell viability was measured by staining cells with PI followed by flow cytometric analysis. **b** Western blot analysis showing TRP53 expression in each variant of the isogenic *Eμ-Myc* lymphoma cells. Probing for β-ACTIN was used as a loading control. **c** The *Eμ-Myc* lymphoma cells with the different TRP53 states were treated for 24 h with the MDM2 inhibitor, nutlin-3a (10 μM), a potent activator of wt TRP53. Cell viability was measured by staining cells with PI followed by flow cytometric analysis. **d** The *Eμ-Myc* lymphoma cells with the different TRP53 states were treated for 48 h with the indicated concentrations of APR-246. Cell viability was measured by staining cells with PI followed by flow cytometric analysis. *N* = 3 independent experiments for each cell line and cell variant. Data are presented as mean ± S.D.

The *Eµ-Myc* transgenic mouse is a useful pre-clinical model of human aggressive B cell lymphomas, such as Burkitt Lymphoma (BL) [33]. Similar to human BL, spontaneous *Trp53* mutations arise in ~20–30% of mouse *Eµ-Myc* lymphomas [34]. Therefore, it was possible to compare the sensitivity to APR-246 of *Eµ-Myc* lymphoma-derived cell lines expressing wt TRP53 with those expressing mutant TRP53. Treatment with APR-246 did not alter the levels of wt TRP53 or mutant TRP53 protein in these lymphoma cells (data not shown). Surprisingly, the *Eμ-Myc* lymphoma cell lines expressing wt TRP53 were more susceptible to killing induced by APR-246 compared to *Eμ-Myc* lymphoma cells expressing mutant TRP53 (Fig. 1a). This prompted us to generate isogenic wt or mutant TRP53-ablated variants of *Eμ-Myc* lymphoma cells to further interrogate the specific roles of wt and mutant TRP53 in APR-246 induced killing of malignant cells. The wt *Trp53* gene was deleted in the AH15A and AF47A *Eμ-Myc* lymphoma cell lines that are naturally wt for *Trp53* by using CRISPR/Cas9 (Fig. 1b, Supplementary Table S1). Mutant *Trp53* was deleted in the MRE412 (R246Q) and EMRK1172 (D278N) *Eμ-Myc* lymphoma cell lines that had spontaneously acquired a *Trp53* mutation during their development (Fig. 1b, Supplementary Table 1). Western blot analysis of the engineered cell lines confirmed the efficient removal of wt or mutant TRP53, respectively (Fig. 1b). Cell viability measurement after treatment with the MDM2 inhibitor nutlin-3a, a potent activator of wt TP53 [35], confirmed the different TRP53 states in each of these cell line variants. The wt TRP53 expressing cells were, as expected, sensitive to nutlin-3a and the TRP53 deficient as well as the mutant TRP53 expressing lymphoma cells were profoundly resistant to nutlin-3a (Fig. 1c). Cell viability assays revealed that concentrations of APR-246 that can be achieved in human patients in ongoing clinical trials (personal communication Lars Abrahmsen, Aprea) were able to kill *Eμ-Myc* lymphoma cell lines that were TRP53 WT, TRP53 mutant and TRP53 deficient. (Fig. 1d). Interestingly, the wt TRP53 *Eμ-Myc* lymphoma cells were consistently more sensitive to low doses of APR-246 than their TRP53-deficient counterparts.

**Fig. 2:**
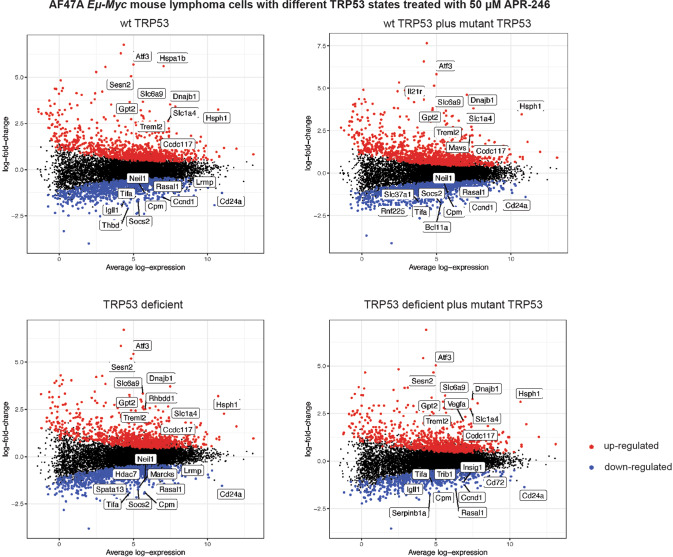
Treatment with APR-246 causes the same changes in gene expression in *Eμ-Myc* mouse lymphoma cells regardless of their TRP53 state. Mean-difference plots for the RNA sequencing differential gene expression analysis comparing four different TRP53 states of isogenic background *Eμ-Myc* mouse lymphoma cells. The x-axis shows the average gene log expression while the y-axis shows gene log2 fold change after treatment with APR-246. Boxes indicate top 10 genes that are significantly up-regulated or down-regulated, respectively, after treatment with 50 μM APR-246 in the *Eμ-Myc* lymphoma cells with the four different TRP53 states for 8 h.

Moreover, we used the AH15A and AF47A wt *Trp53 Eμ-Myc* lymphoma cell lines to generate isogenic background cells with all four possible TRP53 states, namely wt TRP53, wt TRP53 alongside R270H mutant TRP53 (expressed from a retroviral vector), wt *Trp53* knockout and wt *Trp53* knockout alongside R270H mutant TRP53 (expressed from a retroviral vector) (Supplementary Fig. S1a). The four different TRP53 states were confirmed by Western blotting (Supplementary Fig. S1b) and treatment of the cells with nutlin-3a, where the wt TRP53 expressing cells were highly sensitive and the TRP53 knockout cells as well as the R270H mutant TRP53 expressing cells profoundly resistant (Supplementary Fig. S1c). The isogenic *Eμ-Myc* lymphoma cells of all four TRP53 states could be readily killed by treatment with APR-246, although contrary to predictions from previous reports [36], the wt *Trp53* knockout plus R270H mutant TRP53 cells were more resistant than the other variants (Supplementary Fig. S1d). RNAseq analysis revealed that treatment with APR-246 induced up-regulation and down-regulation of the same groups of genes in these *Eμ-Myc* lymphoma cells impacting the same cellular processes, regardless of their TRP53 state (Fig. 2). The expression of *Trp53* mRNA was not altered upon treatment with APR-246 in any of these cell variants (Supplementary Fig. S2a). Most of the top differentially expressed genes were reported to be involved in responses to diverse cellular stresses, including *Atf3*, *Hsp105*, *Gpt2*, *Tifa*, *Ccnd1* and *Socs2* [37–42] (Supplementary Fig. S2b). These findings reveal that mutant TRP53 is dispensable for the response of *Eμ-Myc* mouse lymphoma cells to APR-246.

### Removal of mutant TP53 does not impact APR-246 induced killing of human cancer derived cell lines

**Fig. 3:**
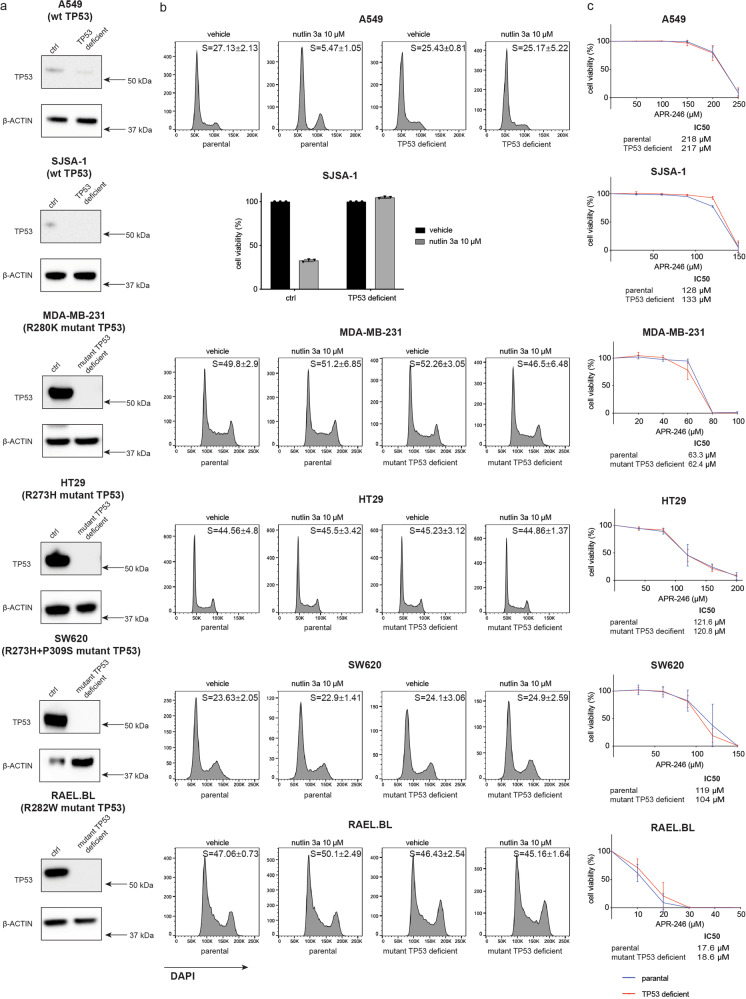
APR-246 can kill human cancer derived cell lines independent of mutant TP53. **a** Western blot analysis showing a reduction of either wt TP53 or mutant TP53 protein expression in each cancer cell line due to the efficient inactivation of the *TP53* gene by CRISPR/Cas9. Probing for β-ACTIN was used as a loading control. **b** The indicated human cancer cell lines with the different TP53 states were treated with nutlin-3a (10 μM). Cell viability was measured by staining cells with PI followed by flow cytometric analysis, and cell cycling was measured by DAPI staining. **c** APR-246 induced killing of mutant TP53 expressing parental cancer cell lines and their mutant TP53 deleted derivatives. Cells were treated with the indicated concentrations of APR-246 and after 2 days cell survival was measured by flow cytometric analysis after staining with PI. *N* = 3 independent experiments for each cell line and cell variant. Data are presented as mean ± S.D.

To further clarify whether the killing of malignant cells induced by APR-246 is dependent on mutant TP53, we used CRISPR/Cas9 technology to generate isogenic background human cancer cell lines of diverse origin that had different TP53 states owing to the removal of endogenous wt TP53 or mutant TP53, respectively (Supplementary Table S1). This included the breast cancer cell line MDA-MB-231 (R280K mutant TP53), the colorectal cancer cell lines HT29 (R273H mutant TP53) and SW620 (R273H and P309S mutant TP53), the Burkitt lymphoma cell line Rael-BL (R282W mutant TP53), the lung cancer cell line A549 (wt TP53) and the osteosarcoma cell line SJSA-1 (wt TP53) [24]. Western blotting confirmed the efficient deletion of mutant TP53 or wt TP53 in each cell line after transduction with Cas9 and a *TP53* specific sgRNA (Fig. 3a). As expected, treatment with nutlin-3a induced either cell cycle arrest or cell death in the tumour cells expressing wt TP53 but not in the TP53 deficient or mutant TP53 expressing cells (Fig. 3b). Cell viability assays revealed that APR-246 was able to kill the paired isogenic cancer cells with similar efficiency, irrespective of their TP53 state (wt vs deleted, mutant *vs* deleted) (Fig. 3c). This demonstrates that APR-246 can kill human tumour cells independent of mutant or wt TP53.

### Low doses of APR-246 kill *Eμ-Myc* lymphoma cells through BAX/BAK-dependent apoptosis

**Fig. 4:**
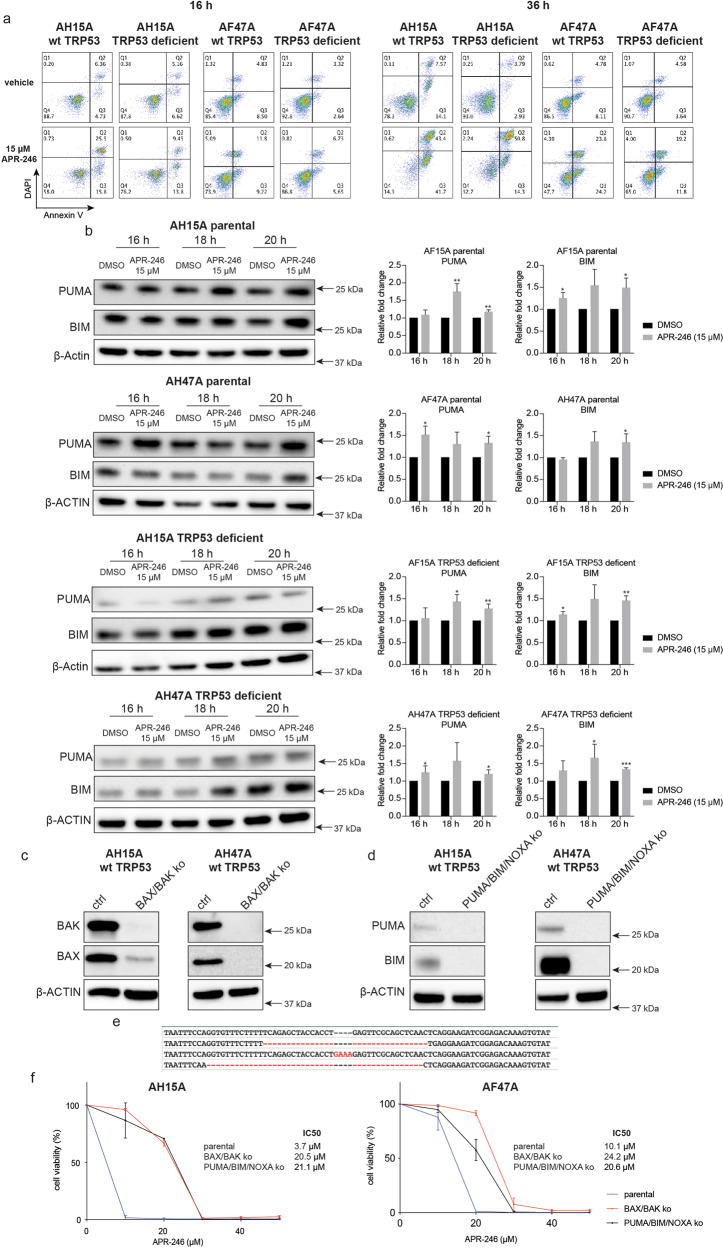
Low doses of APR-246 activate the intrinsic apoptotic cell death pathway in *Eμ-Myc* mouse lymphoma cells. **a** The *Eμ-Myc* lymphoma cell lines indicated, either parental with wt TRP53 or their TRP53 deleted derivatives, were treated for 16 h or 36 h with 15 μM APR-246 (a relatively low dose). The cells were then stained with DAPI plus A647 conjugated Annexin V and examined by flow cytometric analysis. **b** Western blot analysis of the indicated *Eμ-Myc* lymphoma cell lines, either parental with wt TRP53 or their TRP53 deleted derivatives, that had been treated for 16, 18 or 20 h with 15 μM APR-246 (a relatively low dose), showing the expression of PUMA, BIM and β-ACTIN (loading control). **c** Western blot analysis showing the expression of BAX and BAK in the indicated parental *Eμ-Myc* lymphoma cell lines expressing wt TRP53 and their absence in their BAX/BAK double knockout derivatives. Probing for β-ACTIN was used as a loading control. **d** Western blot analysis showing the expression of PUMA and BIM in the indicated parental *Eμ-Myc* lymphoma cell lines expressing wt TRP53 and their PUMA/BIM/NOXA triple knockout derivatives. Probing for β-ACTIN was used as a loading control. **e** NGS sequencing showing the mutations in the *Noxa* gene in the PUMA/BIM/NOXA triple knockout derivatives of the indicated *Eμ-Myc* lymphoma cell line. **f** The indicated *Eμ-Myc* lymphoma cell lines, parental as well as their BAX/BAK double knockout and PUMA/BIM/NOXA triple knockout derivatives, were treated for 48 h with the indicated concentrations of APR-246. Cell viability was measured by staining cells with PI followed by flow cytometric analysis. *N* = 3 independent experiments for each cell line and cell variant. Data are presented as mean ± S.D.

To try to identify the cell death pathway(s) that are triggered by APR-246, we performed live cell imaging in *Eμ-Myc* mouse lymphoma cells expressing wt TRP53 after treatment with a relatively low dose of APR-246 (15 μM). Hallmarks of apoptosis were observed, such as nuclear condensation and fragmentation as well as cell shrinkage (Supplementary Fig. S3 and Supplementary Movie 1). Studies using the isogenic *Eµ-Myc* lymphoma cell lines of the four different TRP53 states and staining with Annexin V plus DAPI followed by flow cytometric analysis revealed that these lymphoma cells were undergoing apoptosis upon APR-246 treatment regardless of their TRP53 state. Interestingly, considerably more apoptosis was seen in the lymphoma cells expressing wt TRP53 compared to TRP53 mutant or deficient lymphoma cells (Fig. 4a).

Western blot analysis showed that treatment with APR-246 caused an increase in the levels of the pro-apoptotic BH3-only protein PUMA, a critical initiator of apoptosis mediated by TRP53 [43], in both the wt TRP53 *Eµ-Myc* lymphoma cells and their TRP53 deficient derivatives, although to a greater extent in the former (Fig. 4b). The pro-apoptotic BH3-only protein BIM was increased upon treatment with APR-246 to a similar extent in both the wt TRP53 and the TRP53 deficient lymphoma cells (Fig. 4b). Using CRISPR/Cas9 we engineered wt TRP53 *Eµ-Myc* lymphoma cells to either lack all three BH3-only proteins, BIM, PUMA and NOXA (collectively required to initiate DNA damage-induced killing of *Eμ-Myc* lymphoma cells [44]) or BAX and BAK the essential effectors of apoptosis [45] (Fig. 4c–e). The combined absence of PUMA, BIM plus NOXA (verified by Western blotting or DNA sequencing: Fig. 4d and e) protected these cells from low doses of APR-246 to a similar extent as combined loss of BAX and BAK (Fig. 4f). These results were consistent with the data from the RNAseq analyses performed in the *Eµ-Myc* lymphoma cells with the four different TRP53 states, showing that only the genes for PUMA, BIM and NOXA were induced upon APR-246 treatment but not genes for the other BH3-only proteins (Supplementary Fig. S2c). These results demonstrate that relatively low concentrations of APR-246 kill *Eµ-Myc* lymphoma cells through BAX/BAK mediated apoptosis by induction of the BH3-only proteins PUMA, NOXA and BIM that can be induced in either a TRP53-dependent or TRP53-independent manner.

### Necroptosis contributes to APR-246 induced killing in certain cancer cell lines

**Fig. 5:**
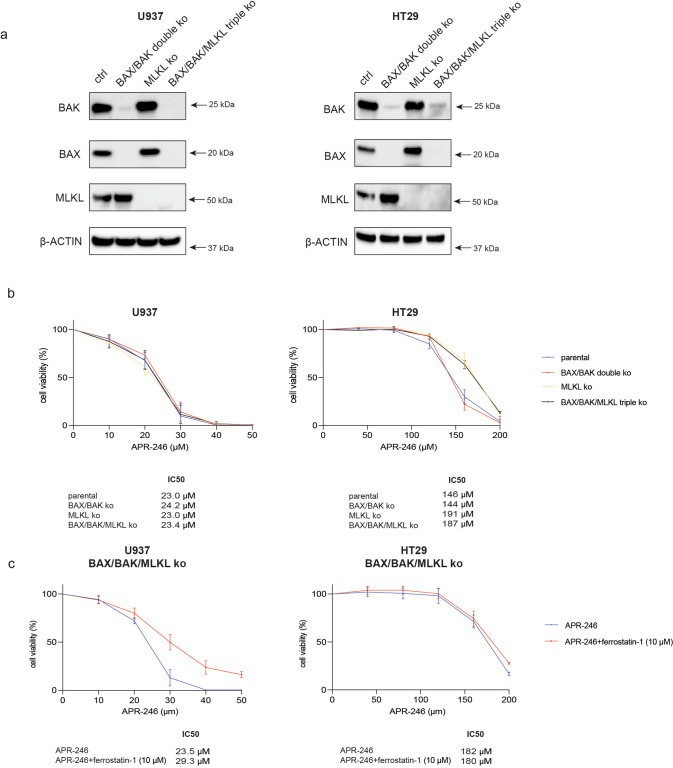
Necroptosis and ferroptosis contribute to APR-246 induced killing in certain human cancer derived cell lines. **a** Western blot analysis of the U937 human histiocytic lymphoma cell line and the HT26 human colorectal cancer cell line: parental, BAX/BAK double knockout, MLKL knockout or BAX/BAK/MLKL triple knockout, showing staining for BAX, BAK, MLKL and β-ACTIN (loading control). **b** The indicated variants of the U937 and HT29 cancer cell lines were treated for 48 h with the indicated concentrations of APR-246. Cell viability was measured by staining with PI followed by flow cytometric analysis. **c** BAX/BAK/MLKL triple knockout derivatives of U937 and HT29 cells were treated for 48 h with the indicated concentrations of APR-246 alone or together with 10 μM ferrostatin-1 (an inhibitor of ferroptosis). Cell viability was measured by staining cells with PI followed by flow cytometric analysis. *N* = 3 independent experiments for each cell line and cell variant. Data are presented as mean ± S.D.

We found that the combined absence of BAX and BAK did not protect the human cancer cell lines A549 and HCT116 from APR-246 induced killing (Supplementary Fig. S4). Therefore, we investigated whether necroptosis, a lytic form of programmed cell death [46], might be involved in APR-246 induced killing of these cancer cells. Necroptosis is induced by RIPK1 and RIPK3 and executed by MLKL [47]. The human U937 histiocytic lymphoma cell line and the HT29 colon cancer cell line (Supplementary Table 1: U937 (TP53 deficient) and HT29 (R273H)) were selected for these studies since they express RIPK1, RIPK3 and MLKL and are able to undergo necroptosis [20, 48]. CRISPR/Cas9 was used to render these cells deficient for MLKL, the essential executioner of necroptosis [46]. Furthermore, BAX and BAK were removed from the parental cells and from their MLKL deficient derivatives (Fig. 5a). In these cells a cocktail of BH3-mimetic compounds targeting the pro-survival proteins BCL-2, BCL-XL, BCL-W and MCL-1 was used to induce apoptosis and the combination of TNFα, a SMAC mimetic plus the broad-spectrum caspase inhibitor IDN-6556 (termed TSI) were used to induce necroptosis. As expected, the intrinsic apoptosis and necroptosis pathways were both profoundly inhibited in the BAX/BAK/MLKL triple knockout U937 and HT29 cells (Supplementary Fig. S5a, b). Notably, the combined absence of BAX and BAK did not protect these two human cancer cell lines from APR-246, even using relatively low doses of this drug (Fig. 5b). This demonstrates that apoptosis is not required for APR-246 induced killing in these two cancer cell lines. Interestingly, the blockage of necroptosis provided significant, albeit relatively minor, protection from APR-246 in HT29 cells but not in U937 cells (Fig. 5b). The combined absence of BAX, BAK and MLKL did not afford greater protection against APR-246 in HT29 cells than loss of MLKL alone, and it had no effect in U937 cells (Fig. 5b). Moreover, the blockage of necroptosis did not protect BAX/BAK double knockout *Eμ-Myc* mouse lymphoma cells from killing by high doses of APR-246 (Supplementary Fig. S6a, b). These findings show that necroptosis can contribute to APR-246 induced killing in some cancer cells.

### Ferroptosis contributes to APR-246 induced killing in certain cancer cell lines

Ferroptosis is a lytic form of programmed cell death that is associated with the abnormal accumulation of lipid peroxides [49]. Ferrostatin, an inhibitor of ferroptosis [50], provided significant, albeit minor, protection against higher doses of APR-246 in BAX/BAK/MLKL triple knockout U937 cells (Fig. 5c), but no protection was observed in HT29 cells. This difference between these two human cancer cell lines may be due to the fact that only U937 cells are prone to ferroptosis, for example caused by 1 μM RSL-3, an inducer of ferroptosis, whereas HT29 cells were not killed by RSL-3 (Supplementary Fig. S7) [51]. Inhibitors of ferroptosis did not protect *Eμ-Myc* mouse lymphoma cells from killing by APR-246 (Supplementary Fig. S6b). Of note, Ferrostatin provided greater protection against APR-246 in U937 cells lacking BAX/BAK or lacking BAX/BAK plus MLKL compared to those lacking only MLKL or the parental cells (Fig. 5c and Supplementary Fig. S8). These findings indicate that apoptosis and ferroptosis cooperate in APR-246 induced killing of U937 cells, but not in HT29 cells or *Eμ-Myc* lymphoma cells. However, BAX/BAK/MLKL triple knockout U937 cells treated with ferrostatin still died after treatment with intermediate doses (40 or 50 mM) of APR-246 (Fig. 5c). These findings reveal that APR-246 can induce ferroptosis to kill certain cancer cells and this can occur alongside apoptosis and necroptosis. However, APR-246 can also induce other cell death processes since cancer cells lacking the critical effectors of apoptosis as well as necroptosis and treated with an inhibitor of ferroptosis can still be effectively killed by APR-246.

### NINJ1 mediates APR-246 induced plasma membrane rupture in malignant cells regardless of which form of programmed cell death is engaged

**Fig. 6:**
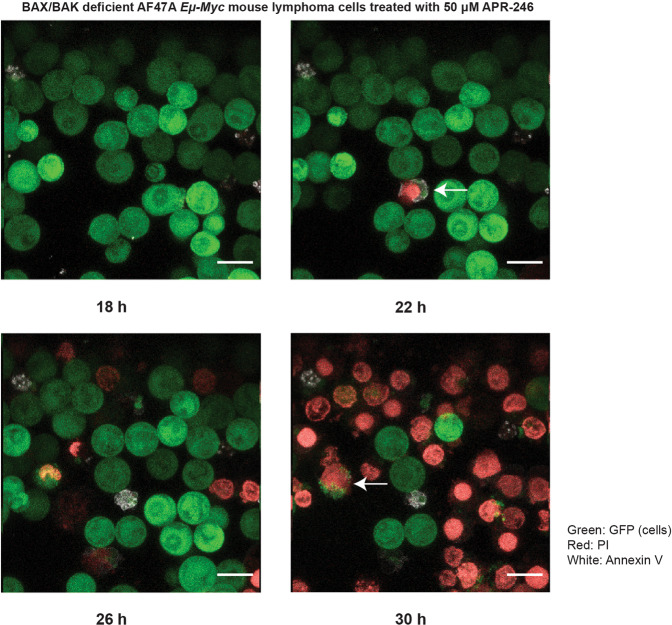
High doses of APR-246 induce cell lysis but not apoptosis in *Eμ-Myc* mouse lymphoma cells. BAX/BAK double knockout *Eμ-Myc* lymphoma cells were treated with a relatively high dose (50 μM) of APR-246 and examined by microscopy. Arrows point to cells undergoing lytic cell death. Green: GFP; Red: PI; White: Annexin V. Magnification: ×1000; scale bar: 10 μm.

Relatively high doses of APR-246 had the ability to kill not only the parental but also the BAX/BAK double knockout *Eμ-Myc* mouse lymphoma cells (Fig. 4f). This demonstrates that higher doses of APR-246 can kill cancer cells through non-apoptotic cell death processes. Live cell imaging was performed on the BAX/BAK double knockout *Eμ-Myc* lymphoma cells after treatment with a relatively high dose (50 μM) of APR-246 to examine the nature of this cell death. Unlike the parental cells, the BAX/BAK double knockout *Eμ-Myc* lymphoma cells did not die upon treatment with a low dose of APR-246 since they cannot undergo apoptosis. At the higher dose the BAX/BAK double knockout *Eμ-Myc* lymphoma cells showed plasma membrane lysis but no nuclear condensation or cell shrinkage (Fig. 6, Supplementary Movie 2).

**Fig. 7:**
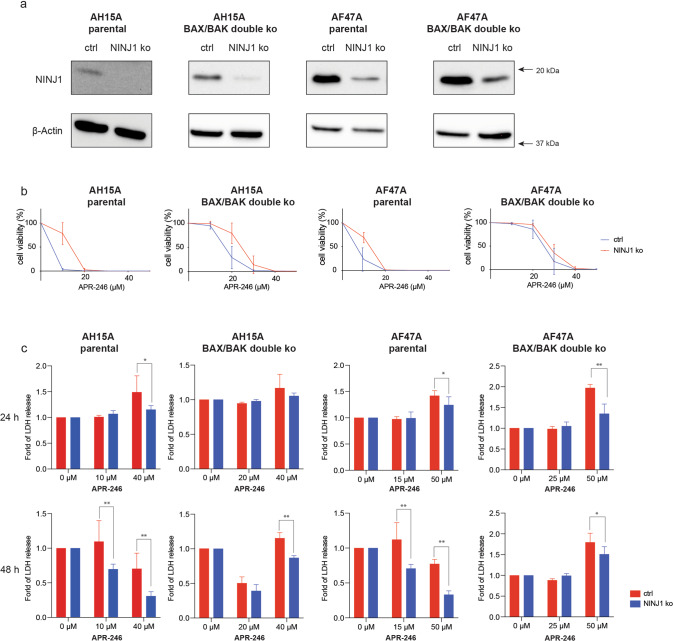
NINJ1 mediates APR-246 induced lysis in *Eμ-Myc* mouse lymphoma cells. **a** Western blot analysis showing the deletion of NINJ1 in the indicated *Eμ-Myc* lymphoma cell lines, either parental or their BAX/BAK double knockout derivatives. **b** The indicated *Eμ-Myc* lymphoma cell lines were treated for 48 h with the indicated concentrations of APR-246. Cell viability was measured by staining cells with PI followed by flow cytometric analysis. **c** The indicated *Eμ-Myc* lymphoma cell lines were treated for 24 h or 48 h with the indicated concentrations of APR-246. LDH release was measured using the CytoTox Non-Radioactive Cytotoxicity assay. *N* = 3 independent experiments for each cell line and cell variant. Data are presented as mean ± S.D.

The cell surface protein NINJ1 was reported to be a mediator of plasma membrane rupture in diverse programmed cell death processes and even in un-programmed necrosis [52]. Since high doses of APR-246 induced lytic death in *Eμ-Myc* lymphoma cells, even those deficient for BAX and BAK, we examined whether NINJ1 was involved in the APR-246 induced lysis of these cells by deleting NINJ1 using CRISPR/Cas9 in both wt and BAX/BAK deficient *Eµ-Myc* lymphoma cells (both expressing wt TRP53). Western blotting confirmed reduced NINJ1 expression in the parental and BAX/BAK double knockout *Eμ-Myc* lymphoma cells that had been transduced with the *Ninj1* specific sgRNA (Fig. 7a). Cell viability assays revealed that the absence of NINJ1 provided only minor protection from APR-246 induced killing, as determined by PI staining and flow cytometric analysis, in both the parental as well as the BAX/BAK double deficient cells (Fig. 7b). Importantly, however, at low doses of APR-246 the loss of NINJ1 significantly reduced the release of LDH, a marker of plasma membrane rupture, from the wt cells over 48 h (Fig. 7c). Little LDH release was seen in the BAX/BAK double knockout lymphoma cells at the relatively low dose of APR-246, regardless of whether they expressed or lacked NINJ1 (Fig. 7c), because these cells are resistant to this treatment (Fig. 4f). In contrast, at higher doses (50 μM) of APR-246, NINJ1 loss caused a reduction in LDH release from both the wt and BAX/BAK deficient lymphoma cells (Fig. 7c), consistent with both cell types undergoing a lytic form of cell death under these conditions. Live cell imaging confirmed that upon treatment with high doses (50 μM) of APR-246, NINJ1-deficient as well as BAX/BAK/NINJ1 triple knockout *Eμ-Myc* lymphoma cells did not show signs of cell lysis, although cell death could still be observed as evidenced by staining with PI (Supplementary Fig. S9 and Supplementary movies 3 and 4). The NINJ1-deficient cells displayed cell enlargement, consistent with a previous report [52].

APR-246 could also induce lytic cell death in certain human cancer derived cell lines (Fig. 5). So, we examined whether NINJ1 was involved in the APR-246 induced ferroptosis and necroptosis by deletion of NINJ1 in HT29 and U937 parental cells as well as their BAX/BAK double knockout derivatives. NGS sequencing confirmed the efficient deletion of *Ninj1* gene in each cancer cell line transduced with a *Ninj1* specific sgRNA (Supplementary Fig. S10a). Cell viability assays revealed that the removal of NINJ1 did not have marked impact on APR-246 induced killing (Supplementary Fig. S10b) but significantly reduced the release of LDH (Supplementary Fig. S10c) in both the parental cancer cells and their BAX/BAK double knockout derivatives. These findings demonstrate that NINJ1 is involved in the rupture of the plasma membrane in cells treated with APR-246 regardless of whether they are dying by apoptosis or a non-apoptotic process.

### Responses of non-transformed cells to APR-246

**Fig. 8:**
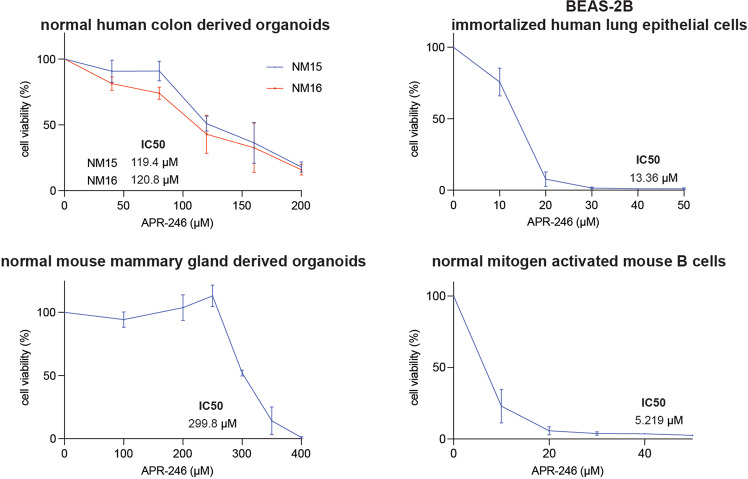
Response of non-transformed cells to APR-246. The indicated non-transformed cells were treated for 48 h with APR-246 and cell viability was examined either by using the Celltiter Glo assay (organoids and non-transformed lung epithelial cells) or by PI staining followed by flow cytometric analysis (activated B lymphocytes). *N* = 3 independent experiments for each cell type. Data are presented as mean ± S.D.

There are still only limited data on the impact of APR-246 on non-transformed cells. Such data are critical for the further development of this compound in clinical trials. We examined the sensitivity of mitogen activated normal mouse B cells, normal mouse mammary gland derived organoids, normal human colon derived organoids and immortalised (but not transformed) BEAS-2B human bronchial epithelial cells to APR-246. Mitogen activated mouse B cells and human BEAS-2B lung epithelial cells were rather sensitive to APR-246 while mouse mammary glands and human colon organoids were relatively resistant (Fig. 8). This reveals that different types of non-transformed cells exhibit different sensitivities to APR-246 (Fig. 8). Although mouse mammary gland organoids were more refractory to APR-246 than human breast cancer derived cell lines, the responses of the other non-transformed cells to APR-246 were comparable to those of their malignant counterparts. In the case of the lung, the non-transformed cells were even more vulnerable than the lung cancer cells (compare data from Figs. 1d, 3c and 8). These findings inform on a potential therapeutic window for the use of APR-246 in cancer treatment.

## Discussion

Since APR-246 is being tested in several clinical trials for cancer therapy (ClinicalTrials.gov Identifier: NCT02999893, NCT03931291, NCT04214860 and NCT04383938), it is important to define the mechanism(s) by which this compound kills malignant cells and its impact on non-transformed cells from healthy tissues. This knowledge may help identify which anti-cancer agents might cooperate with APR-246 to inhibit tumour growth. We generated panels of isogenic tumour cell lines with different TP53/TRP53 states to determine whether APR-246 induced killing of malignant cells depends on mutant TP53/TRP53, as previously reported [53]. Four human cancer derived cell lines and murine *Eμ-Myc* lymphoma cell lines, which had undergone selection for mutations in *TP53/Trp53* during their development, were selected and the endogenous mutant *TP53/Trp53* gene was inactivated using CRISPR/Cas9 to generate mutant TP53/TRP53 deleted derivatives. There were no significant differences in the response to APR-246 between the parental cells and their mutant TP53 deleted derivatives. These findings demonstrate that APR-246 can kill malignant cells independent of mutant TP53. Furthermore, we generated *Eμ*-Myc mouse lymphoma cell lines with all four possible TRP53 states, wt TRP53, TRP53 deficient, mutant TRP53 and wt TRP53 alongside mutant TRP53. RNAseq analysis demonstrated that treatment with APR-246 induced very similar changes in gene expression in Eμ-Myc lymphoma cells of all four possible TRP53 states. Analysis of these lymphoma cell variants revealed that loss of wt TRP53 as well as enforced expression of mutant TRP53 reduced the killing of these lymphoma cells by low doses of APR-246.

In previously published studies which reported that only mutant TP53/TRP53 expressing cells were sensitive to APR-246 [54, 55], the wt or mutant TP53 expressing human cancer cell lines tested were derived from different tumours and thus had distinct genetic backgrounds with different oncogenic driver mutations. It could therefore not be excluded that the sensitivity or resistance of these malignant cells to APR-246 treatment was due to the tumour type and/or differences in drivers of malignant transformation rather than the presence or absence of mutant TP53. In contrast, our isogenic background tumour cell lines differ only in their TP53/TRP53 states. This allowed us to exclude that differences in genes other than those encoding wt or mutant TP53/TRP53 could have impacted the response to APR-246. Hence, our studies provide compelling evidence that mutant TP53/TRP53 is not critical for APR-246 induced killing of malignant cells.

We found that APR-246 can activate several different cell death pathways depending on the type of cancer cell line. This appears consistent with the fact that MQ, the active metabolite of APR-246, can covalently bind to and modify hundreds of proteins in cells [56]. Low doses of APR-246 killed *Eμ-Myc* lymphoma cells through the activation of the BAX/BAK-dependent intrinsic apoptotic pathway involving the initiators of apoptosis PUMA, NOXA and BIM. These BH3-only proteins were induced in these lymphoma cells upon treatment with APR-246 through both TRP53-dependent and TRP53-independent processes. This explains why the wt TRP53 *Eμ-*Myc lymphoma cells were more sensitive to APR-246 than their TRP53 deficient counterparts. Prior work on APR-246 also indicated that this compound can induce apoptosis, but this was ascribed to its ability to act on mutant TP53 [54]. It was also reported that in human oesophageal carcinoma cells APR-246 can induce the expression of NOXA through activation of the TP53 related transcription factor TP73 [57]. It is possible that in the *Eμ*-*Myc* mouse lymphoma cells, TP73 was activated to induce the expression of the pro-apoptotic proteins BIM, PUMA and NOXA to thereby induce apoptosis.

Moreover, we found that APR-246 was able to induce necroptosis in HT29 human colon cancer cells and ferroptosis in U937 human histiocytic lymphoma cells. Consistent with the latter finding, it was previously shown that APR-246 induced killing of human acute myeloid leukaemia (AML) cells could be reduced, although not abrogated, by inhibitors of ferroptosis [58].

It is intriguing that APR-246 can induce distinct forms of cell death in different cancer cell lines. A possible explanation might be that these cells are prone to activating these specific cell death pathways. For example, abnormally high levels of c-MYC, as seen in *Eμ-Myc* lymphoma cells, are known to render cells prone to activating the intrinsic apoptosis pathway [59]. Moreover, we found that U937 cells are more sensitive to inducers of ferroptosis compared to the other cancer derived cell lines that we have tested. We therefore propose that APR-246 kills malignant cells through the cell death pathway that can be most easily activated in that particular cell type.

In conclusion, we have shown that mutant TP53/TRP53 is not required for the ability of APR-246 to kill malignant cells. Thus, in clinical trials of APR-246, patients should not be selected on the basis of bearing tumours that express mutant TP53 but those with wt TP53 or TP53-deficient tumours should also be included. A recent study pointed out that the expression of SLC7A11 is a more reliable determinant to predict the responses of tumour cells to APR-246 than TP53 states [60]. Moreover, our findings that indicate APR-246 can activate a range of programmed cell death pathways suggest that this compound may cooperate with agents that can induce these cell death pathways in specific tumour types. For example, our findings suggest that inducers of the intrinsic apoptotic pathway, such as BH3-mimetic drugs [61], may enhance the killing effects of APR-246 in lymphoid and possibly also myeloid malignancies. These ideas could be helpful in guiding future clinical trials of APR-246 or related compounds. It must, however, be noted that we found that at least in vitro we did not find a substantial difference in sensitivity to APR-246 when comparing several non-transformed cell types with their malignant counterparts. This may indicate that doses of APR-246 that are required to effectively kill cancer cells may exert substantial toxicity to healthy tissues, possibly making it difficult to establish a therapeutic window.

## Supplementary information

Supplemental Material

Supplemental Movies 1-4

uncropped Western blots

reproducibility checklist

## Data Availability

All data presented in this paper will be made available upon request.
