# Correction to: The anti-cancer agent APR-246 can activate several programmed cell death processes to kill malignant cells

**DOI:** 10.1038/s41418-023-01137-w

**Published:** 2023-02-24

**Authors:** Zilu Wang, Huimin Hu, Luuk Heitink, Kelly Rogers, Yue You, Tao Tan, Connie Li Wai Suen, Alex Garnham, Hao Chen, Elizabeth Lieschke, Sarah T. Diepstraten, Catherine Chang, Tianwei Chen, Diane Moujalled, Kate Sutherland, Guillaume Lessene, Oliver M. Sieber, Jane Visvader, Gemma L. Kelly, Andreas Strasser

**Affiliations:** 1 The Walter and Eliza Hall Institute of Medical Research, Melbourne, VIC 3052 Australia; 2 Department of Medical Biology, University of Melbourne, Melbourne, VIC 3052 Australia; 3 Department of pharmacology and Therapeutics, University of Melbourne, Melbourne, VIC 3052 Australia

**Keywords:** Cancer, Cell biology, Biochemistry

Correction to: *Cell Death & Differentiation* 10.1038/s41418-023-01122-3, published online 04 February 2023

The original version of this article contained a mistake. When preparing the revised version of this paper the authors omitted a new co-author by mistake. The correct authorship list should be: Zilu Wang1,2; Huimin Hu1,2; Luuk Heitink1,2; Kelly Rogers1,2; Yue Y0u1,2; Tao Tan1,2; Connie Li Wai Suen1,2; Alex Garnham1,2; Hao Chen1,2; Elizabeth Lieschke1,2; Sarah T Diepstraten1,2; Catherine Chang1; Tianwei Chen1,2; Diane Moujalled1,2; Kate Sutherland1,2; Guillaume Lssene1,2,3; Oliver M Sieber1,2; Jane Visvader1,2; Gemma L Kelly1,2,4 and Andreas Strasser1,2,4.

1 The Walter and Eliza Hall Institute of Medical Research, Melbourne, VIC 3052, Australia, 2 Department of Medical Biology, University of Melbourne, Melbourne, VIC 3052, Australia

3 Department of Pharmacology and Therapeutics, University of Melbourne, Melbourne, VIC 3052, Australia

4 These authors jointly supervised this work: Gemma LKelly, Andreas Strasser. gkelly@wehi.edu.au; strasser@wehi.edu.au

The original article has been corrected.

