# Supplementary material for: The anti-cancer agent APR-246 can activate several programmed cell death processes to kill malignant cells
Source: Cell Death Differ. 2023 Feb 4;30(4):1033–46. doi: 10.1038/s41418-023-01122-3 (PMC10070280; doi:10.1038/s41418-023-01122-3)
Supplement: Supplementary file 1 — Supplemental Material [file 41418_2023_1122_MOESM1_ESM.docx]

**Supplementary Material**

**Supplementary Figure S1 APR-246 can kill *Eµ-Myc* mouse lymphoma cells irrespective of their TRP53 status.**

**a** Diagram showing the generation of isogenic background *Eµ-Myc* mouse lymphoma cell lines with four different TRP53 states: parental wt TRP53, wt TRP53 plus mutant TRP53, TRP53 deficient and TRP53 deficient plus mutant TRP53. **b** Western blot analysis showing TRP53 expression in each variant of the isogenic *Eµ-Myc* lymphoma cell lines. Probing for β-actin was used as a loading control. **c** The *Eµ-Myc* lymphoma cell lines with the four different TRP53 states were treated for 24 h with the MDM2 inhibitor, nutlin-3a (10 μM), a potent activator of wt TRP53. Cell viability was measured by staining cells with PI followed by flow cytometric analysis. **d** The *Eµ-Myc* lymphoma cell lines with the four different TRP53 states were treated for 48 h with the indicated concentrations of APR-246. Cell viability was measured by staining cells with PI followed by flow cytometric analysis. N= 3 independent experiments per cell line. Data are presented as mean ± S.D.


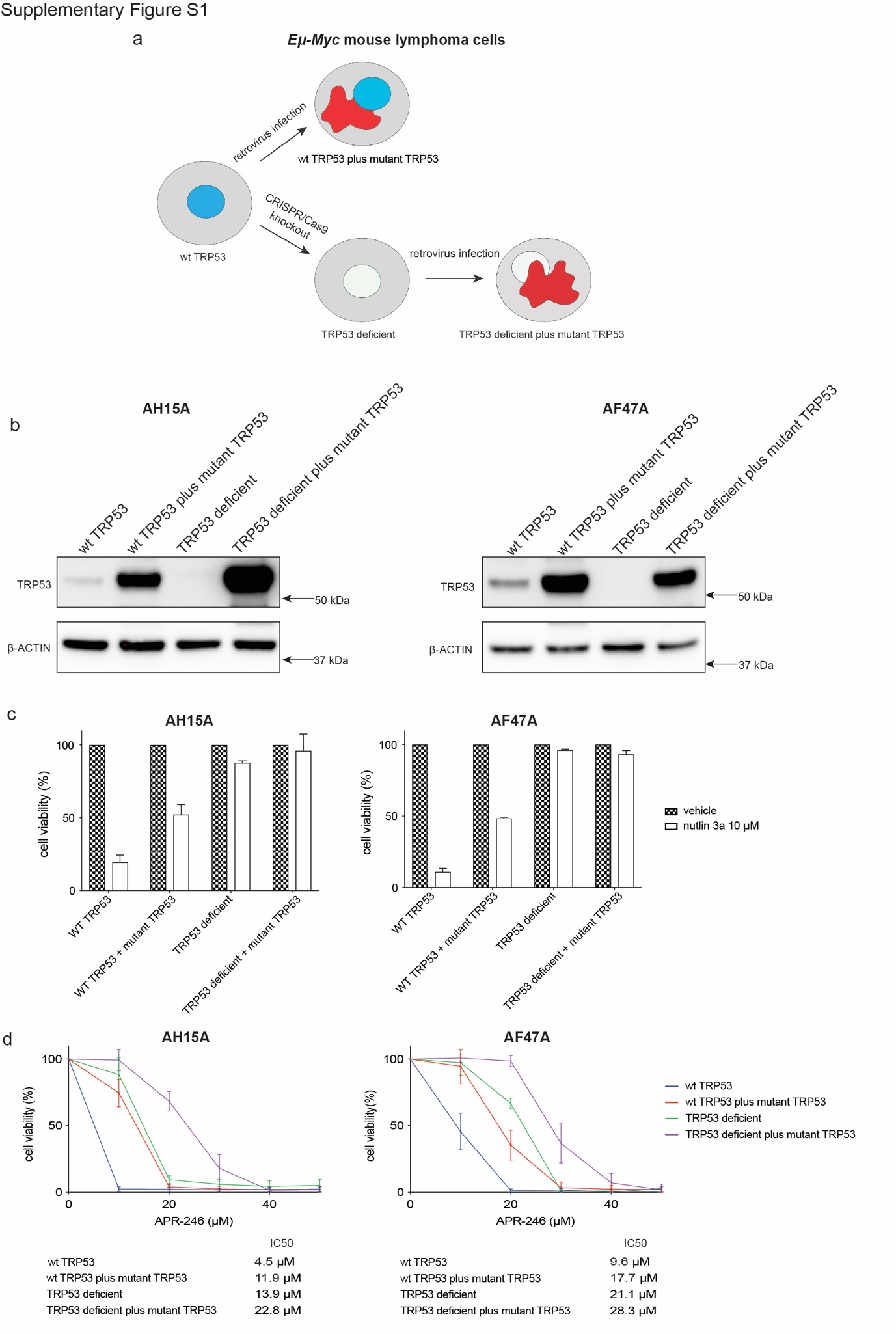


**Supplementary Figure S2 APR-246 induces the expression of the same gene sets in *Eμ-Myc* mouse lymphoma cells of all four possible TRP53 states: wt TRP53 (parental), wt TRP53 alongside mutant TRP53, TRP53 knockout and TRP53 knockout alongside mutant TRP53.**

**a** Glimma interactive MD plot showing that *Trp53* mRNA expression was not altered upon APR-246 treatment. **b** Glimma interactive MD plot showing the top differentially expressed genes in *Eμ-Myc* lymphoma cells of each of the four possible TRP53 states: wt TRP53 (parental), wt TRP53 plus mutant TRP53. TRP53 knockout and TRP53 knockout plus mutant TRP53. **c** The genes for the BH3-only proteins *Puma*, *Bim* and *Noxa*, but not the genes for *Bid*, *Bad*, *Bik* and *Bmf*, are induced in *Eμ-Myc* lymphoma cells of all possible TRP53 states upon treatment with APR-246.


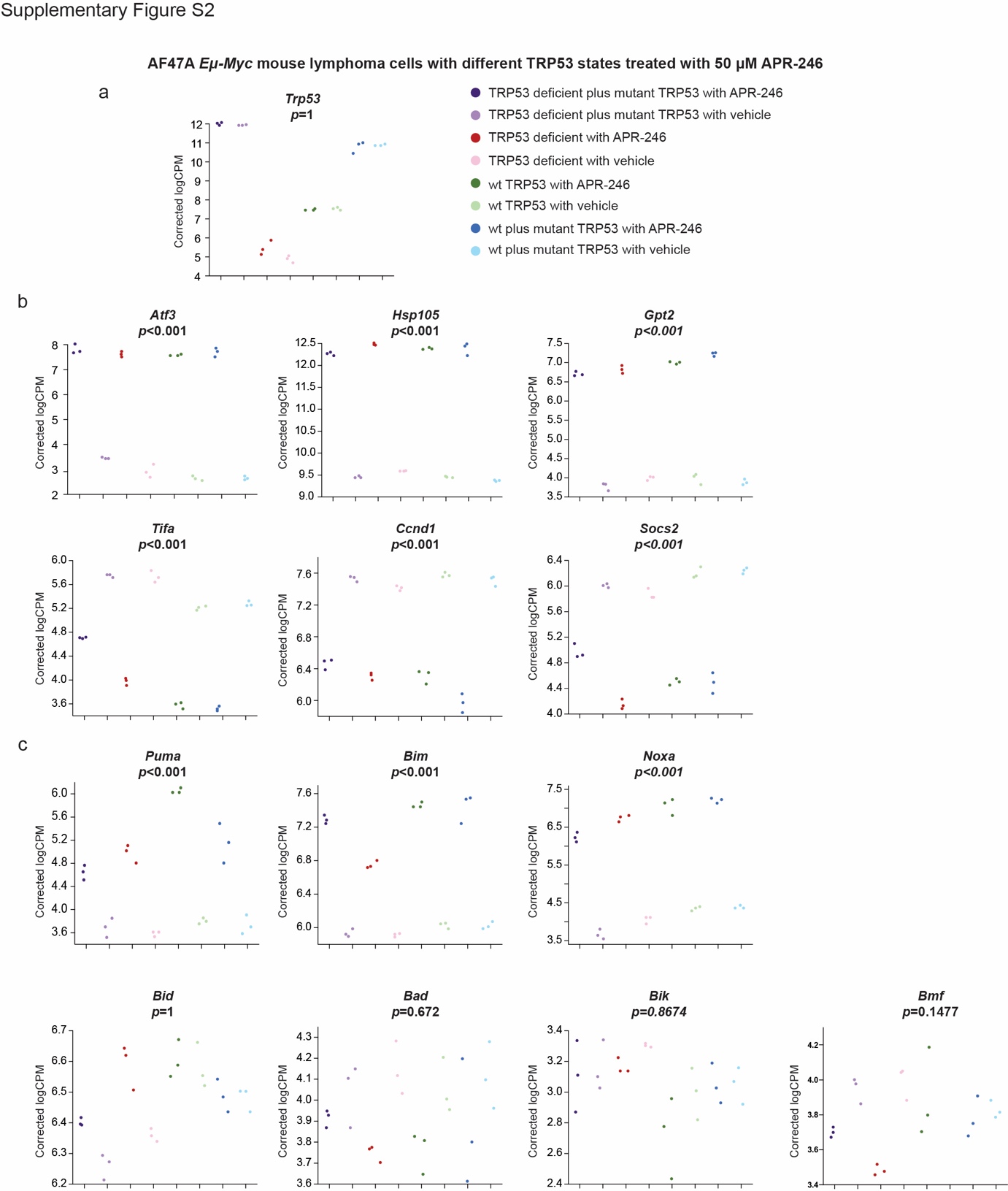


**Supplementary Figure S3 Low doses of APR-246 induce apoptosis in wt *Eμ-Myc* mouse lymphoma cells.**

Parental *Eμ-Myc* lymphoma cells expressing wt TRP53 were treated with a relatively low dose (15 μM) of APR-246. Nuclear fragmentation as well as shrinkage of cells, both hallmarks of apoptosis, were detected by microscopy. Arrows point to cells undergoing apoptosis.

Green: GFP; Red: PI; White: Annexin V. Magnification: 1000x;

scale bar: 10 μm.

**
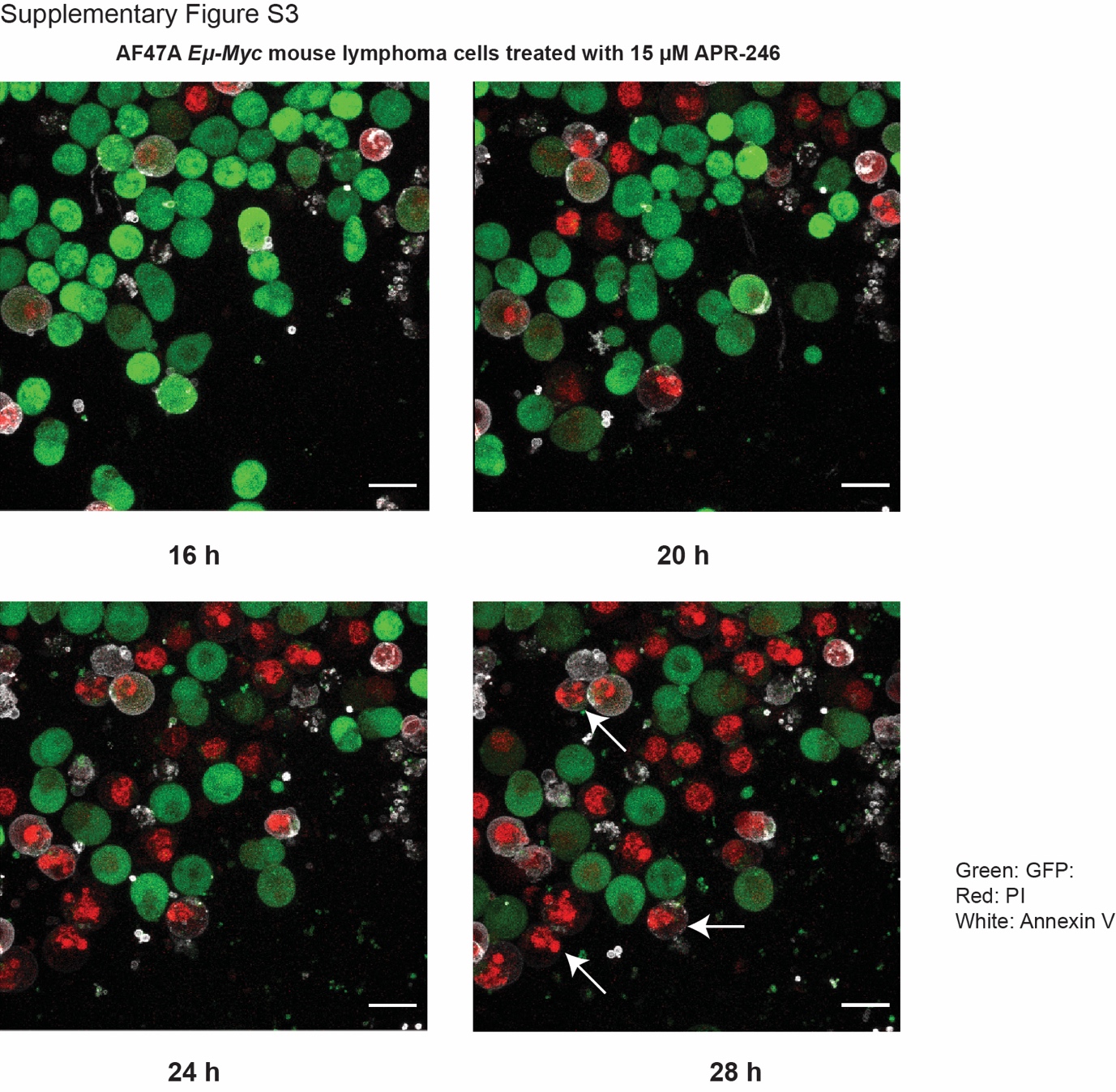
**

**Supplementary Figure S4 APR-246 can kill A549 human lung cancer and HCT116 human colon cancer derived cell lines independent of the activation of the intrinsic apoptosis pathway.**

A549 and HCT116 human lung and colon cancer cells, respectively, either parental or their BAX/BAK double knockout derivatives, were treated for 48 h with APR-246. Cell viability was measured by PI staining and flow cytometric analysis. N= 3 independent experiments for all cell lines and genotypes. Data are presented as mean ± S.D.


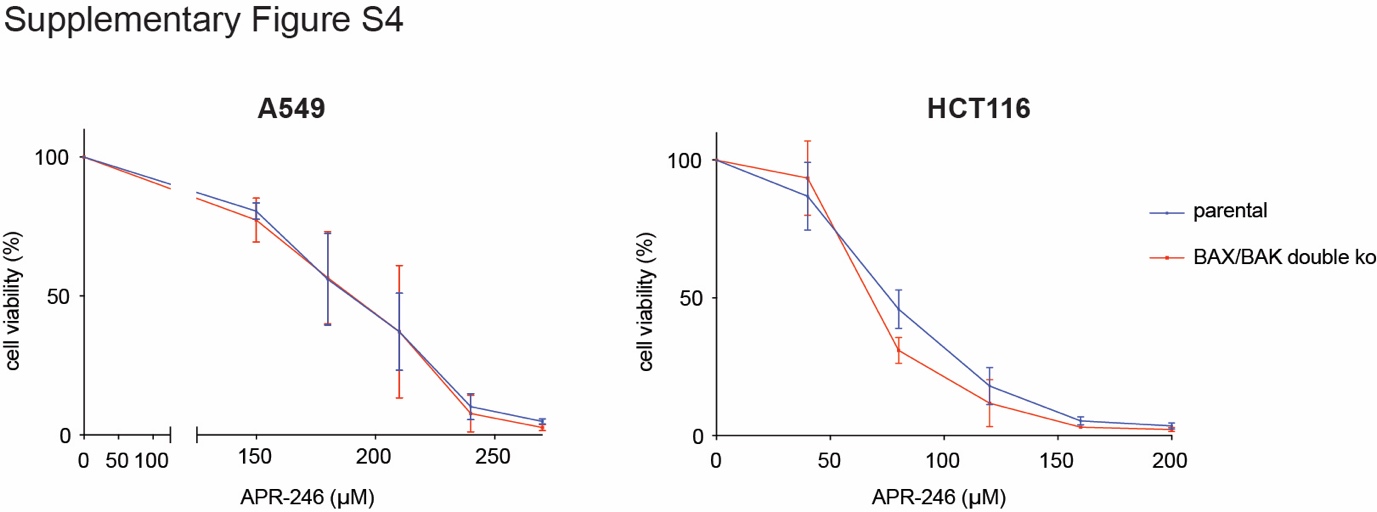


**Supplementary Figure S5 Verification of effective inhibition of apoptosis and necroptosis in U937 human histiocytic lymphoma and HT29 human colon cancer cell lines afforded by the combined deletion of BAX, BAK and MLKL.**

**a** U937 human histiocytic lymphoma cells and HT29 human colon cancer cells, parental or their BAX/BAK/MLKL triple knockout derivatives, were treated for 24 h with either vehicle or a cocktail of three BH3 mimetic compounds: 1 μM ABT-737 (targets BCL-2, BCL-XL and BCL-W) + 1 μM A1331852 (targets BCL-XL) + 1 μM S63845 (targets MCL-1). Cell viability was measured by staining cells with PI followed by flow cytometric analysis. **b** U937 and HT29 cells, parental or their BAX/BAK/MLKL triple knockout derivatives, were treated for 24 h with either vehicle or TSI (100 ng/mL TNFα + 0.5 μM SMAC mimetic Compound A + 5 μM IDN-6556 (broad spectrum caspase inhibitor)). Cell viability was measured by staining cells with PI followed by flow cytometric analysis. N= 3 independent experiments for each cell line and genotype. Data are presented as mean ± S.D.


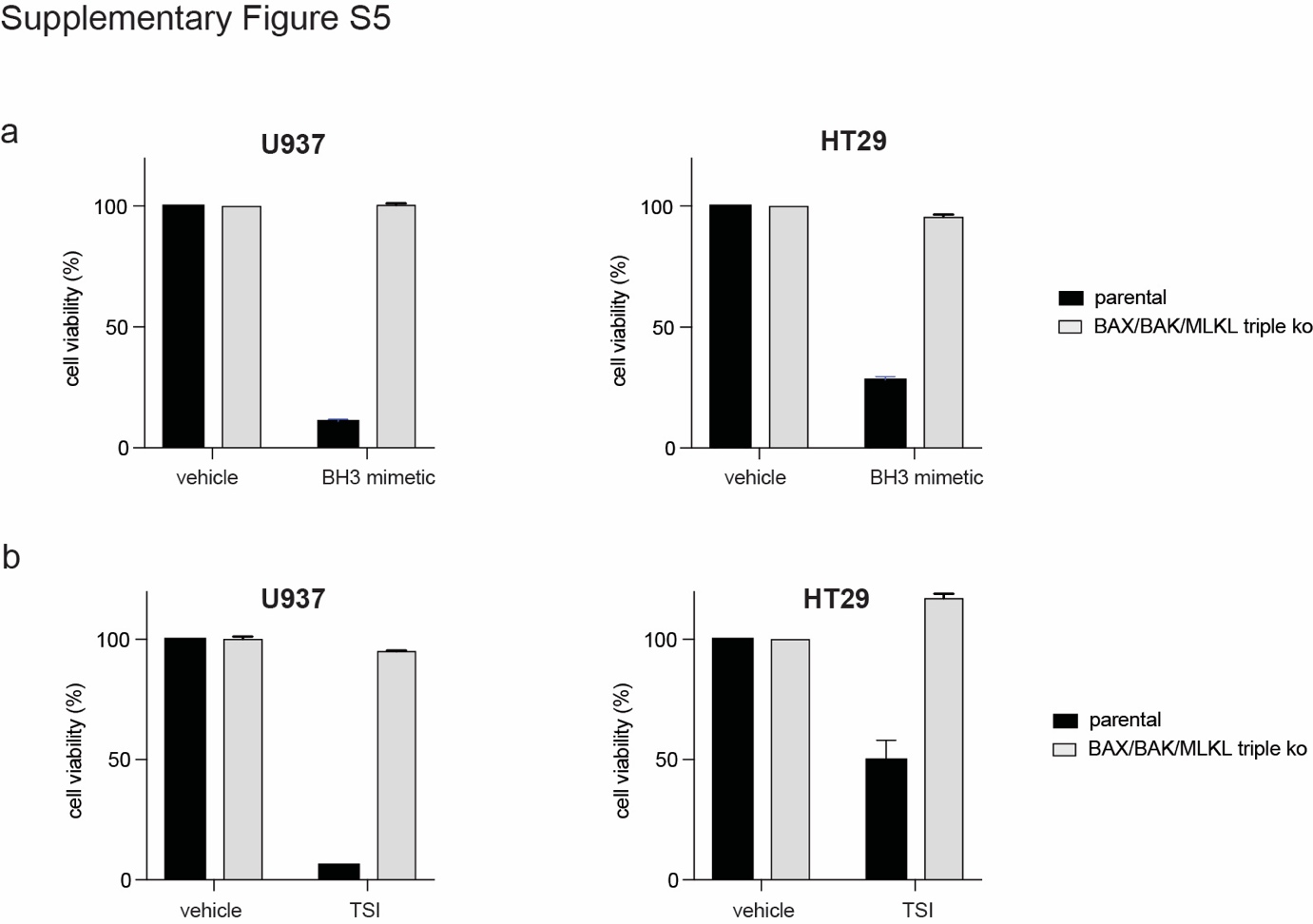


**Supplementary Figure S6 Necroptosis and ferroptosis do not contribute to APR-246 induced killing of *Eµ-Myc* mouse lymphoma cells.**

**a** Western blot analysis showing the deletion of MLKL in BAX/BAK double knockout *Eµ-Myc* lymphoma cells. Probing for β-actin was used as a loading control. The loss of MLKL did not protect these cells from killing by APR-246. **b** Ferrostatin-1 did not protect BAX/BAK/MLKL triple knockout *Eµ-Myc* lymphoma cells from killing by APR-246 although these cells were sensitive to the ferroptosis inducer RSL-3 (1 μM). N= 3 independent experiments. Data are presented as mean ± S.D.


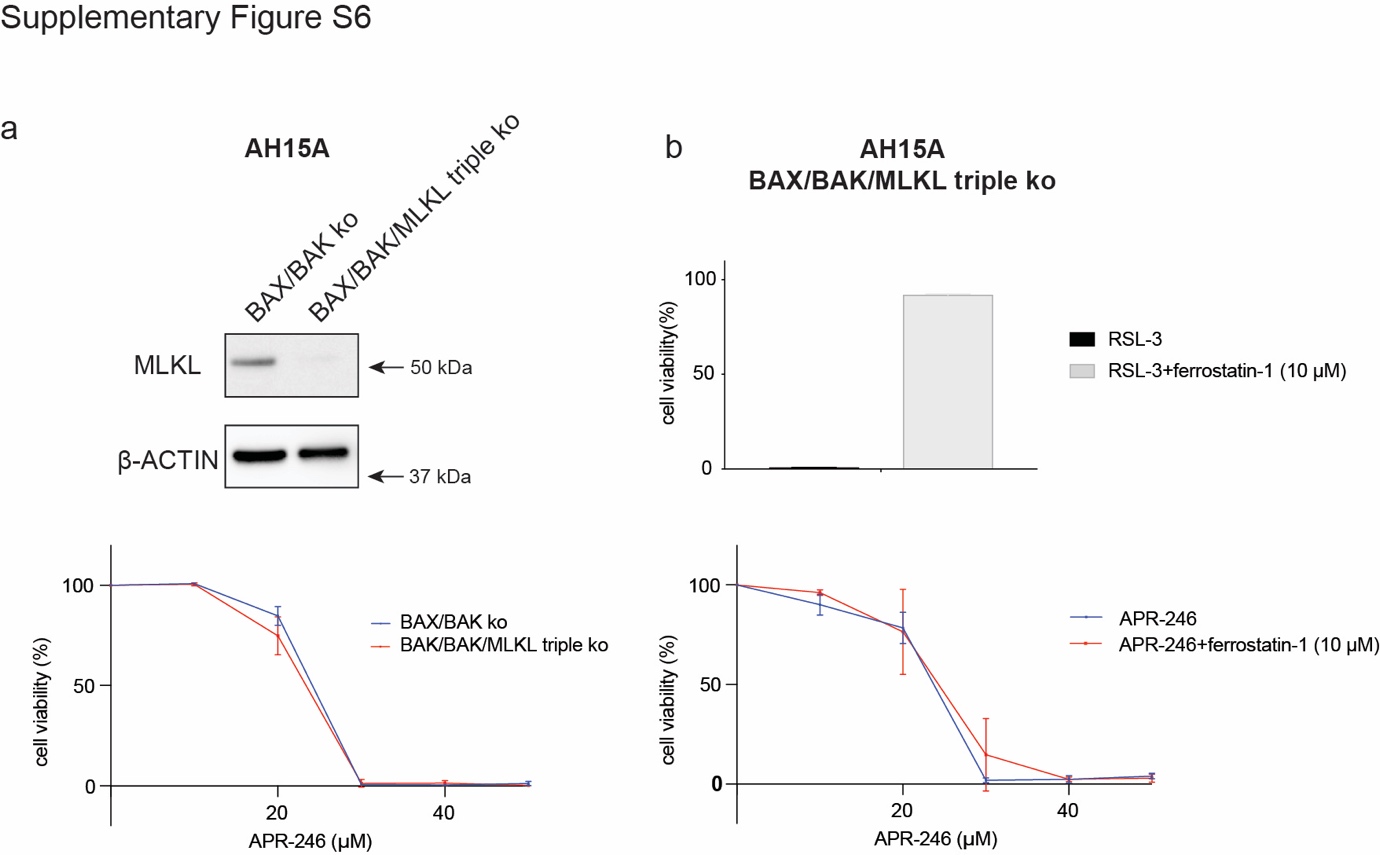


**Supplementary Figure S7 Verification of effective inhibition of ferroptosis in U937 human histiocytic lymphoma and HT29 human colon cancer cell lines by ferrostatin-1.**

BAX/BAK/MLKL triple knockout derivatives of U937 human histiocytic lymphoma cells and HT29 human colon cancer cells were treated for 6 h with either 1 μM RSL-3 (inducer of ferroptosis) alone or 1 μM RSL-3 plus 10 μM ferrostatin-1 (inhibitor of ferroptosis). Cell viability was measured by staining cells with PI followed by flow cytometric analysis. N= 3 independent experiments. Data are presented as mean ± S.D.


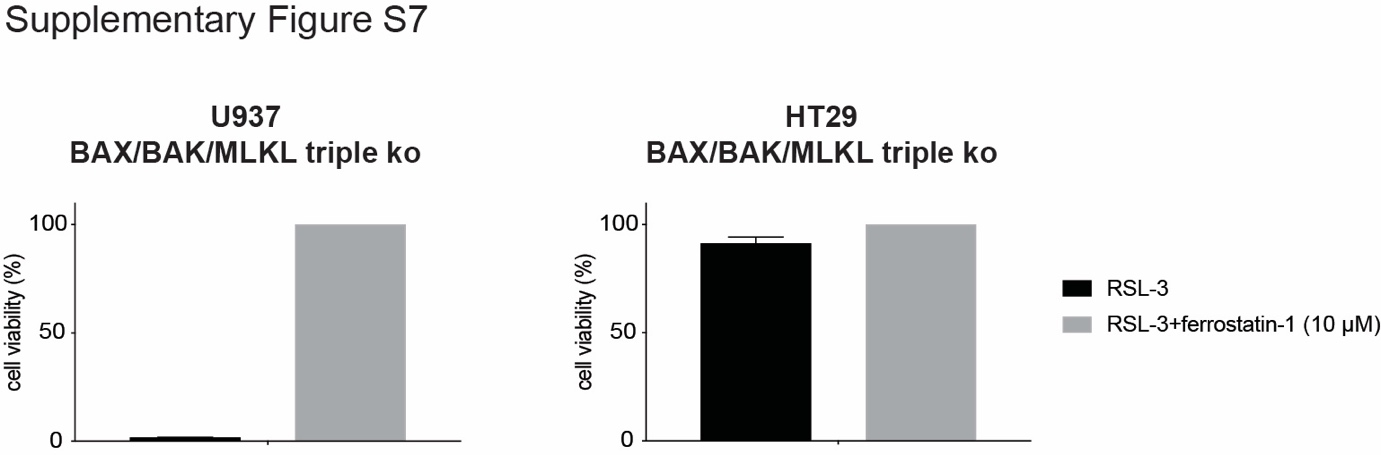


**Supplementary** **Figure S8 BAX/BAK-mediated apoptosis and ferroptosis cooperate in APR-246 induced killing of U937 human histiocytic lymphoma cells.**

U937 human histiocytic lymphoma cells, parental, BAX/BAK double knockout and MLKL knockout, were treated for 48 h with the indicated concentrations of APR-246, either alone or plus the inhibitor of ferroptosis, 10 μM ferrostatin-1. Cell viability was measured by staining cells with PI followed by flow cytometric analysis. N= 3 independent experiments for each cell line and genotype. Data are presented as mean ± S.D.

**Supplementary** **Figure S9 Impact of loss of NINJ1 on the nature of cell death induced by APR-246 in** ***Eμ-Myc* mouse lymphoma cells**

**a** NINJ1 knockout AF47A *Eμ-Myc* mouse lymphoma cells expressing wt TRP53 were treated with a relatively low dose (20 μM) of APR-246 that causes apoptosis in the parental *Eμ-Myc* mouse lymphoma cells. **b** NINJ1/BAX/BAK triple knockout AF47A *Eμ-Myc* mouse lymphoma cells expressing wt TRP53 were treated with a relatively high dose (50 μM) of APR-246 that causes non-apoptotic lytic cell death in the *Eμ-Myc* mouse lymphoma cells. Green: GFP; Red: PI. Magnification: 630X; Scale bar: 10 μm.

White arrows point to cells undergoing swelling after treatment with APR-246.

**
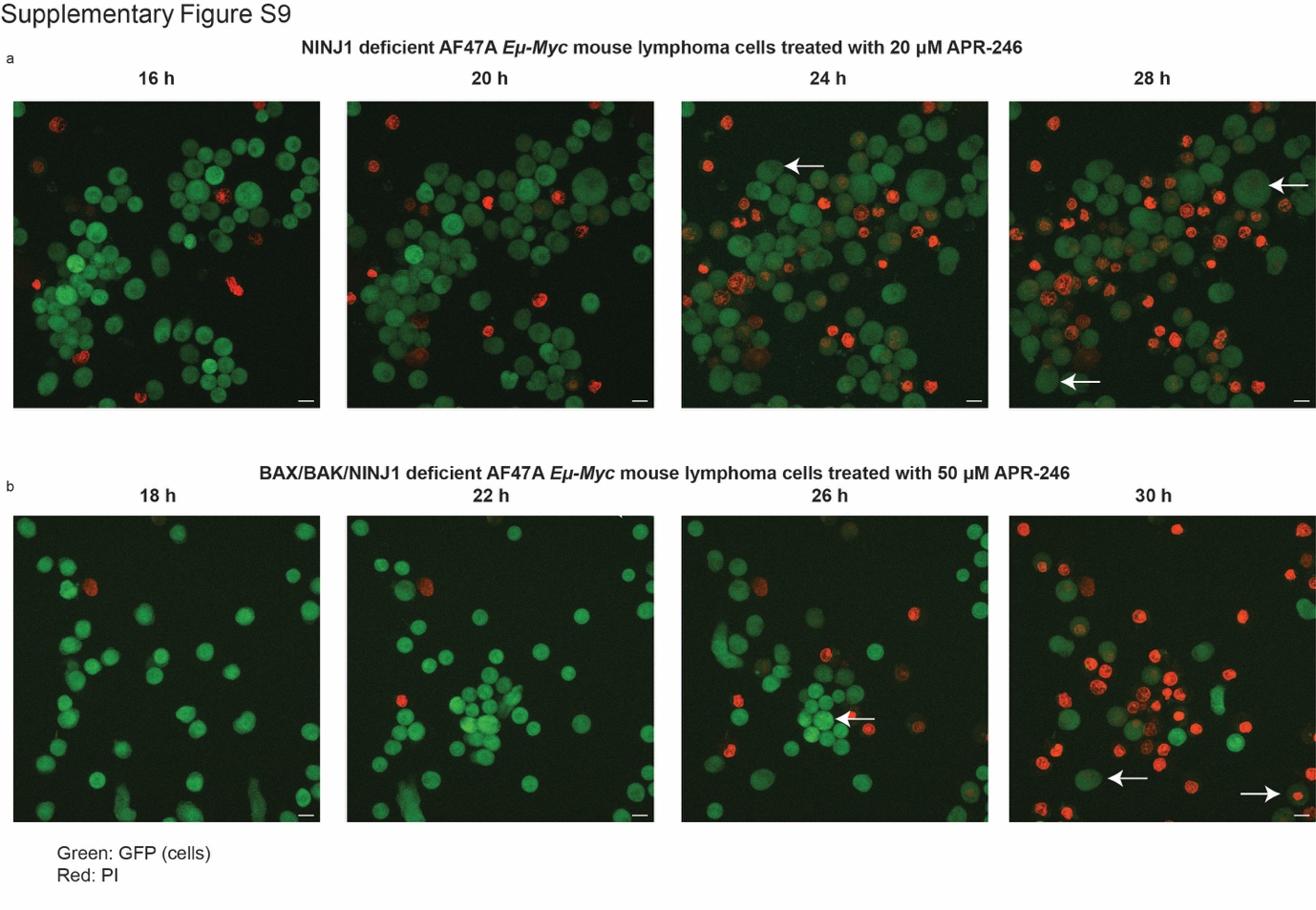
**

**Supplementary** **Figure S10 NINJ1 mediates APR-246 induced lytic cell death in U937 human histiocytic lymphoma cells and HT29 human colon cancer cells.**

**a** NGS sequencing showing the deletion of NINJ1 in U937 human histiocytic lymphoma cells and HT29 human colon cancer cell lines, either parental or their BAX/BAK double knockout derivatives. **b** U937 and HT29 cells of the indicated genotypes (parental; parental with NINJ1 knockout; BAX/BAK double knockout; BAX/BAK double knockout with additional knockout of NINJ1) were treated for 48 h with the indicated concentrations of APR-246. Cell viability was measured by staining cells with PI followed by flow cytometric analysis. **c** U937 and HT29 cells of the indicated genotypes (see above) were treated for 24 h or 48 h with the indicated concentrations of APR-246. LDH release was measured using the CytoTox Non-Radioactive Cytotoxicity assay. N= 3 independent experiments for each cell line and cell variant. Data are presented as mean ± S.D.


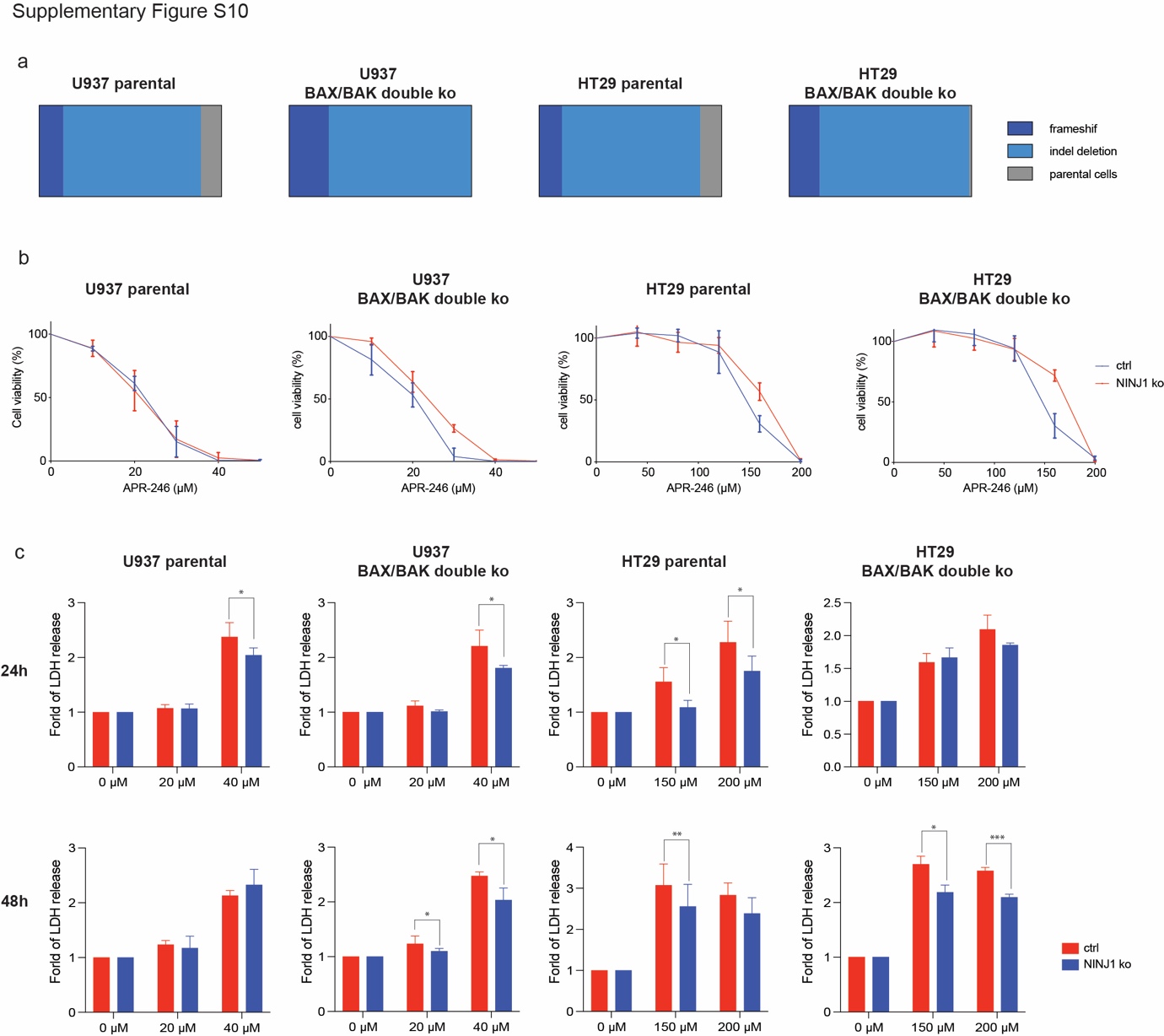


**Supplementary Table S1 Cancer derived cell lines used in this study.**

| Cell lines | Malignancies | TP53  states | Organism |
| --- | --- | --- | --- |
| MDA-MB-231 | Breast cancer | R280K | Human |
| HT29 | Colorectal cancer | R273H | Human |
| SW620 | Colorectal cancer | R273H+P309S | Human |
| Rael.BL | Burkitt lymphoma | R282W | Human |
| A549 | Lung cancer | wt | Human |
| HCT116 | Colorectal cancer | wt | Human |
| SJSA-1 | Osteosarcoma | wt | Human |
| U937 | Histiocytic Lymphoma | null | Human |
| EMRK1172 | *Eμ-Myc* lymphoma | D278N | Mouse |
| MRE412 | *Eμ-Myc* lymphoma | R246Q | Mouse |
| AH15A | *Eμ-Myc* lymphoma | wt | Mouse |
| AF47A | *Eμ-Myc* lymphoma | wt | Mouse |

**Supplementary Table S2 Primers used to sequence mouse *Trp53* exons**

| Primer name | Sequences |
| --- | --- |
| EX4_1 Forward | 5’-GTGACCTATGAACTCAGGAGTCCAGAGCAGAAAGGGACTTGG |
| EX4_1 Reverse | 5’-CTGAGACTTGCACATCGCAGCGCCATAGTTGCCCTGGTAAG |
| EX4_2 Forward | 5’-GTGACCTATGAACTCAGGAGTCTTTTGAAGGCCCAAGTGAAG |
| EX4_2 Reverse | 5’-CTGAGACTTGCACATCGCAGCAGGCATTGAAAGGTCACACG |
| EX5 Forward | 5’-GTGACCTATGAACTCAGGAGTCCGACCTCCGTTCTCTCTCC |
| EX5 Reverse | 5’-CTGAGACTTGCACATCGCAGCGAGGCTGCCAGTCCTAACC |
| EX6 Forward | 5’-GTGACCTATGAACTCAGGAGTCCGGCTTCTGACTTATTCTTGC |
| EX6 Reverse | 5’-CTGAGACTTGCACATCGCAGCCCCTTCTCCCAGAGACTGC |
| EX7 Forward | 5’-GTGACCTATGAACTCAGGAGTCGTAGGGAGCGACTTCACCTG |
| EX7 Reverse | 5’-CTGAGACTTGCACATCGCAGCCCCTAAGCCCAAGAGGAAAC |
| EX8 Forward | 5’-GTGACCTATGAACTCAGGAGTCTCTTACTGCCTTGTGCTGGTC |
| EX8 Reverse | 5’-CTGAGACTTGCACATCGCAGCTGTGGAAGGAGAGAGCAAGA |
| EX9 Forward | 5’-GTGACCTATGAACTCAGGAGTCCCCAAAGTCACCTCTTGCTC |
| EX9 Reverse | 5’-CTGAGACTTGCACATCGCAGCGAGAACCACTGTCGGAGGAG |
| EX10 Forward | 5’-GTGACCTATGAACTCAGGAGTCGGTTGTGTGACCTTGTCCAG |
| EX10 Reverse | 5’-CTGAGACTTGCACATCGCAGCAGCAGGGTGGGGTTTTTATC |
| EX11 Forward | 5’-GTGACCTATGAACTCAGGAGTCCCAAACTGCTAGCTCCCATC |
| EX11 Reverse | 5’-CTGAGACTTGCACATCGCAGCGGCCAGGAACCACTACTCAG |
