# Supplementary material for: The anti-cancer agent APR-246 can activate several programmed cell death processes to kill malignant cells
Source: Cell Death Differ. 2023 Feb 4;30(4):1033–46. doi: 10.1038/s41418-023-01122-3 (PMC10070280; doi:10.1038/s41418-023-01122-3)
Supplement: Supplementary file 3 — uncropped Western blots [file 41418_2023_1122_MOESM3_ESM.pptx]

## Slide 1
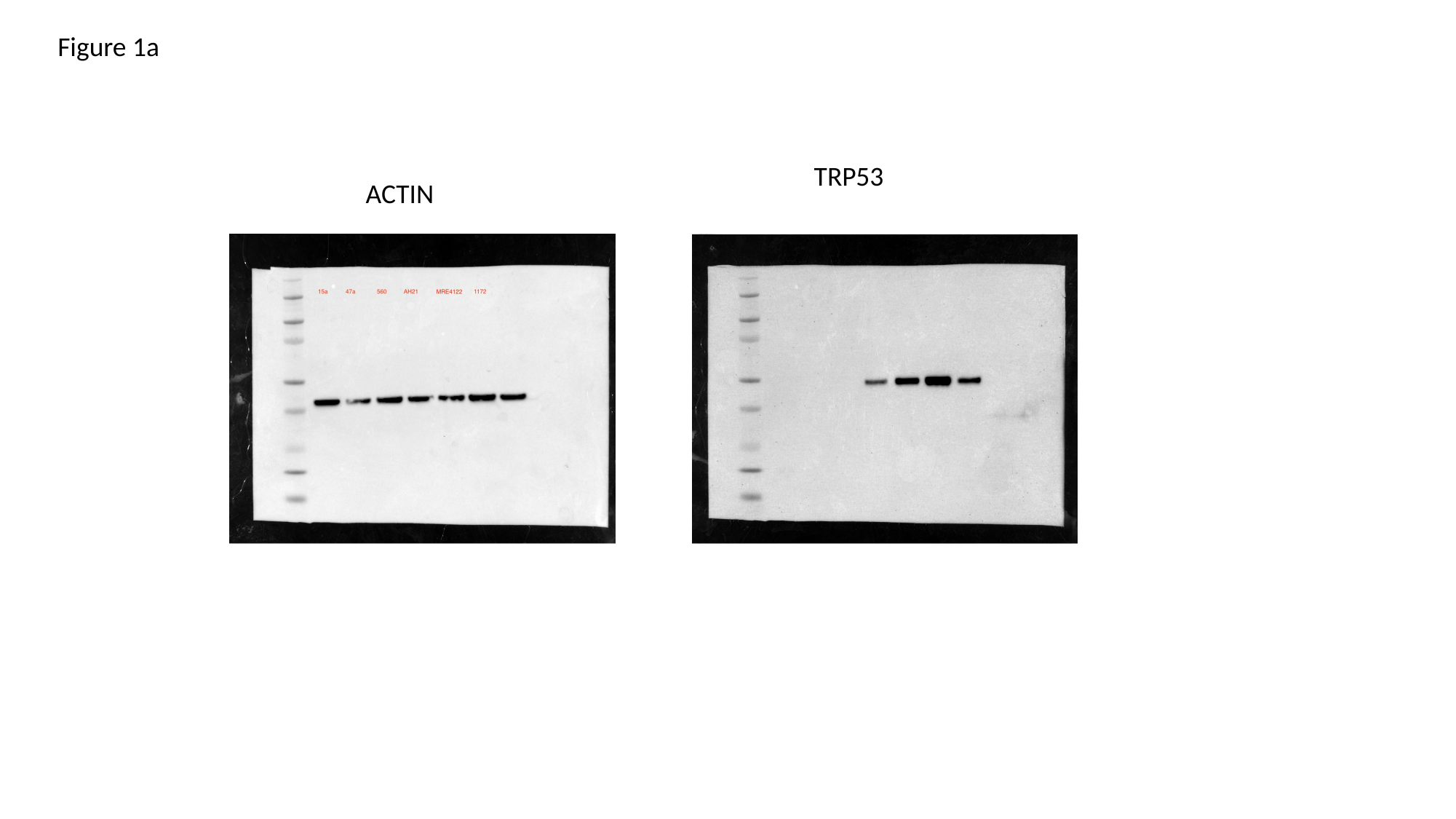

Figure 1a
TRP53
ACTIN

## Slide 2
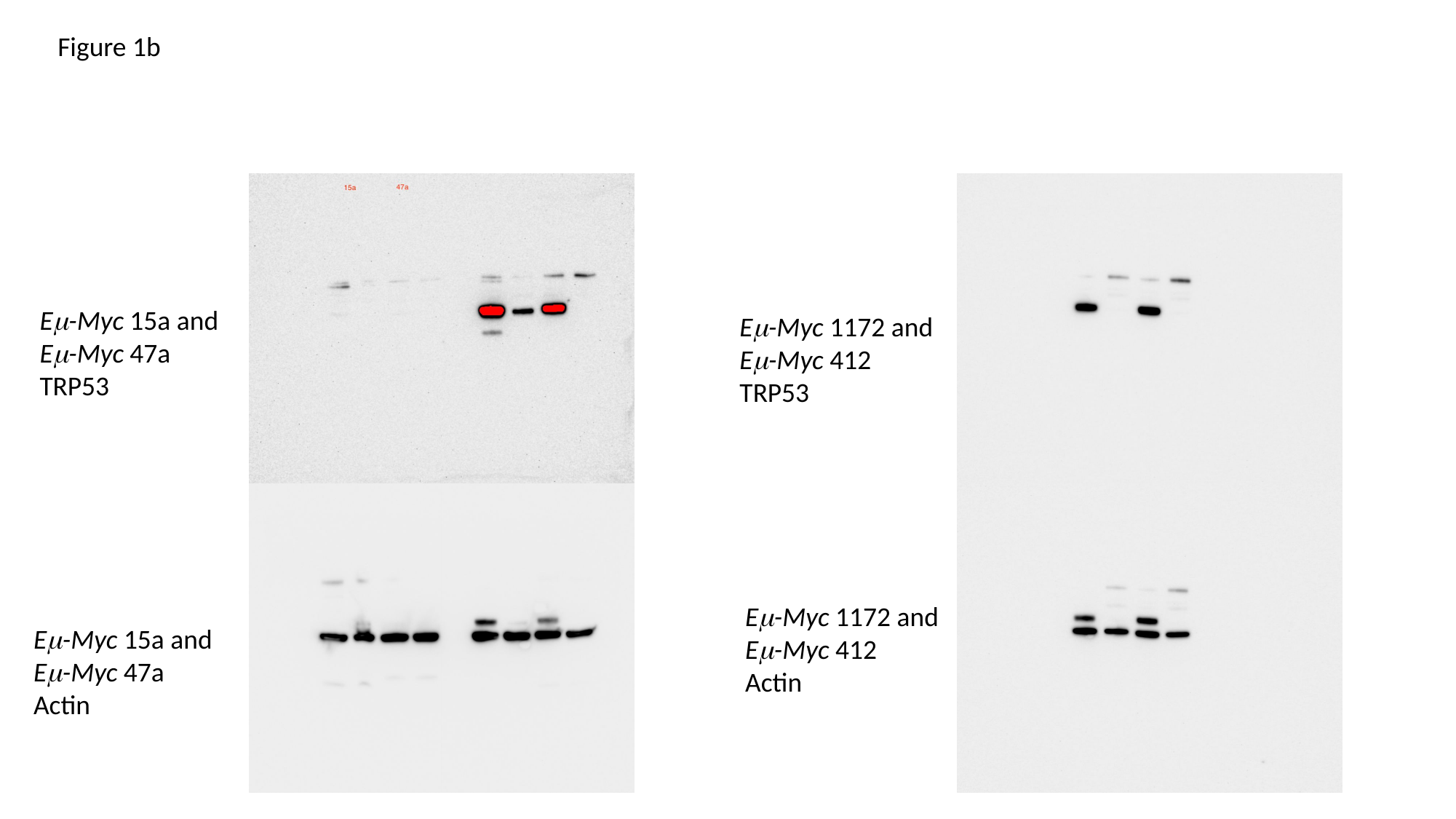

Figure 1b
Em-Myc 15a and
Em-Myc 47a
TRP53
Em-Myc 1172 and
Em-Myc 412
TRP53
Em-Myc 1172 and
Em-Myc 412
Actin
Em-Myc 15a and
Em-Myc 47a
Actin

## Slide 3
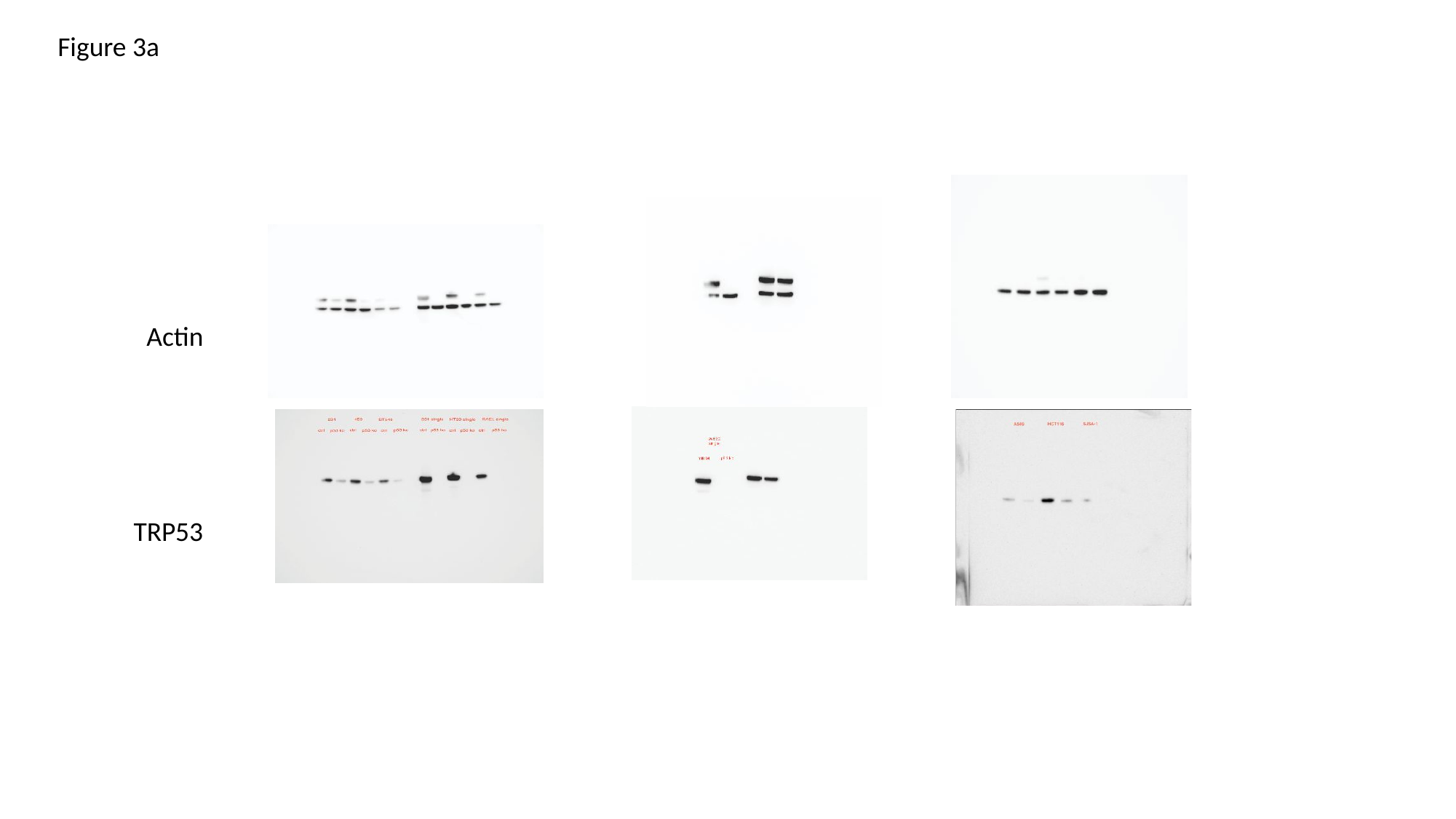

Figure 3a
Actin
TRP53

## Slide 4
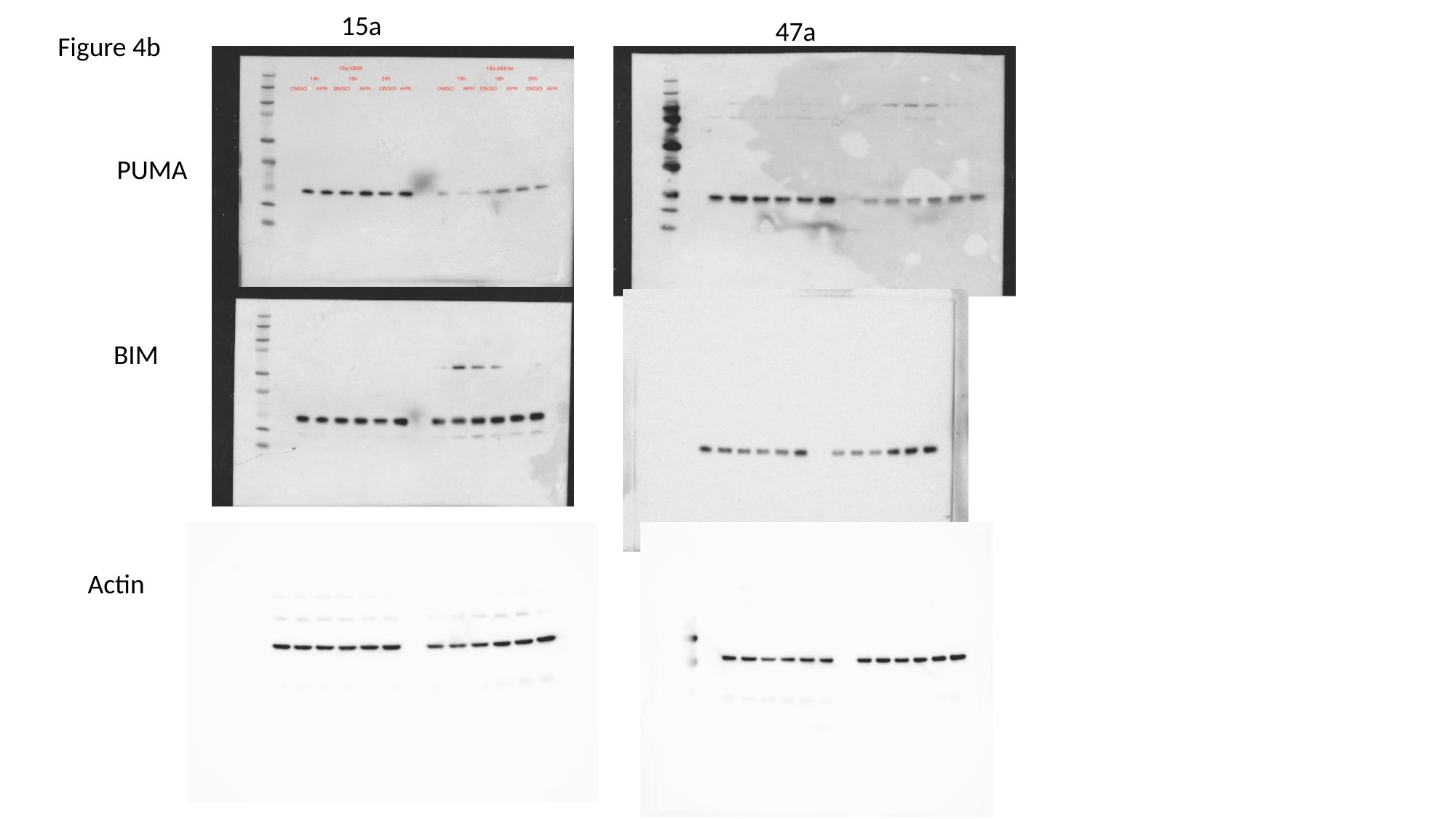

15a
47a
Figure 4b
PUMA
BIM
Actin

## Slide 5
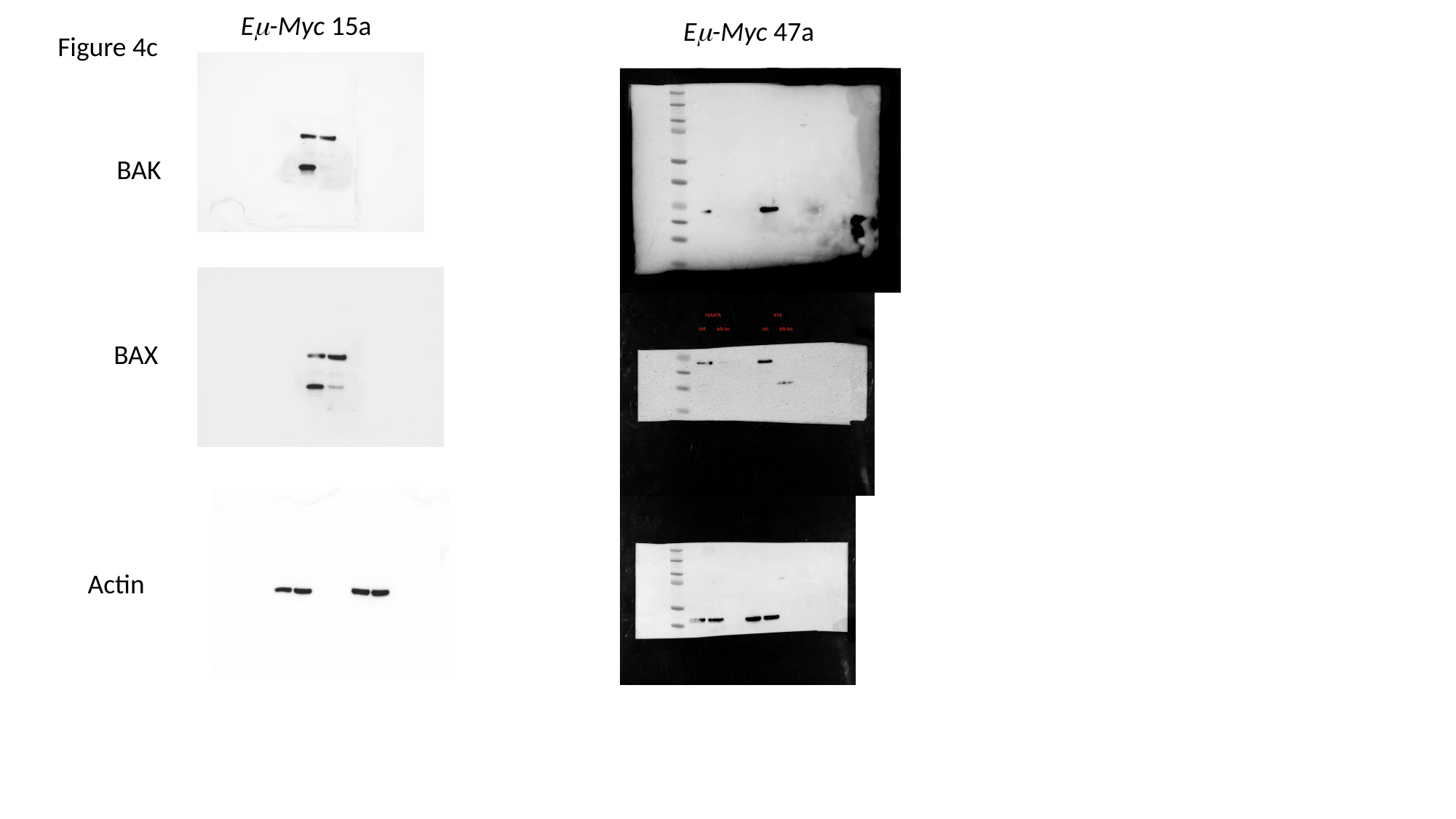

Em-Myc 15a
Em-Myc 47a
Figure 4c
BAK
BAX
Actin

## Slide 6
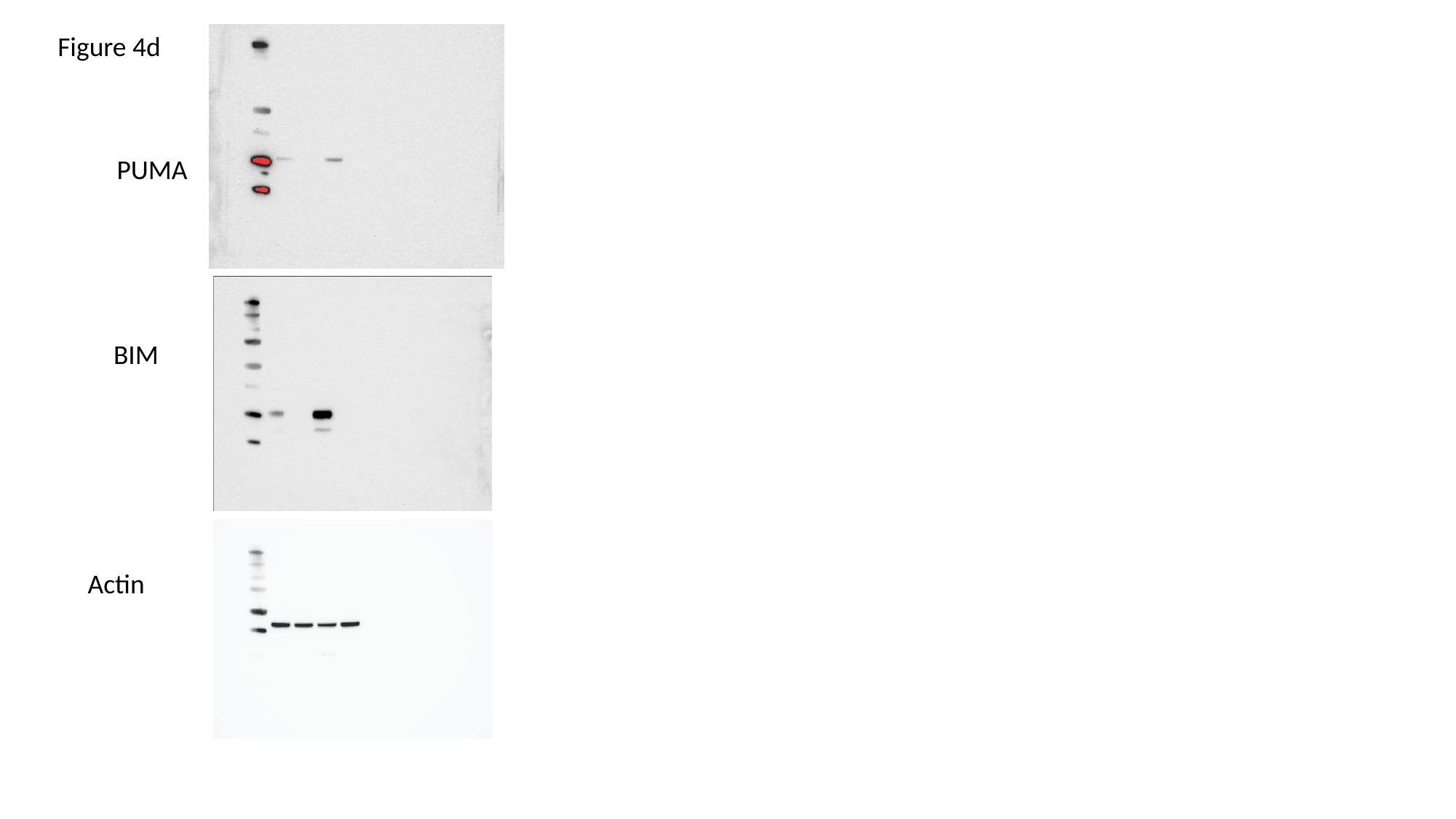

Figure 4d
PUMA
BIM
Actin

## Slide 7
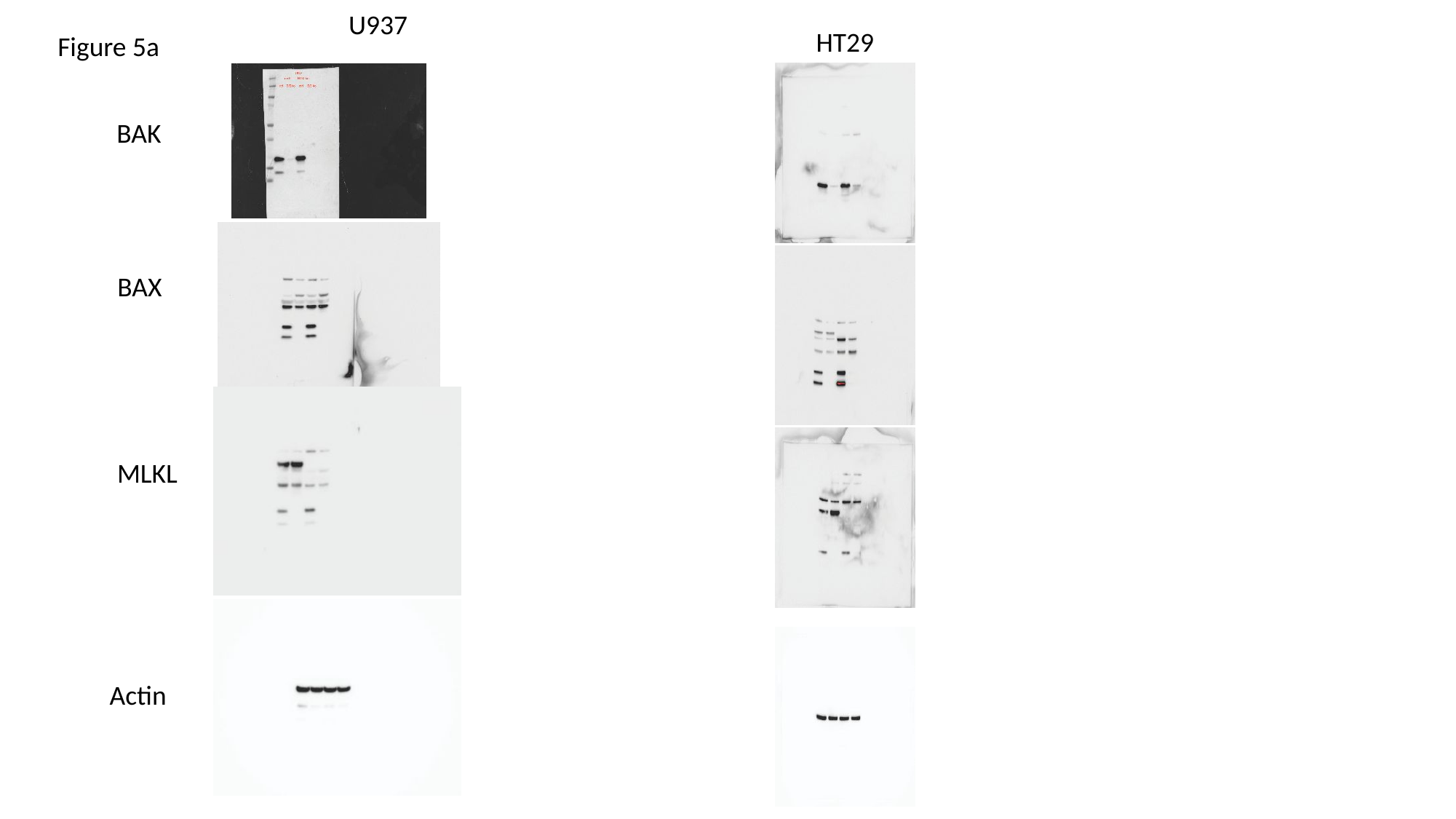

U937
HT29
Figure 5a
BAK
BAX
MLKL
Actin

## Slide 8
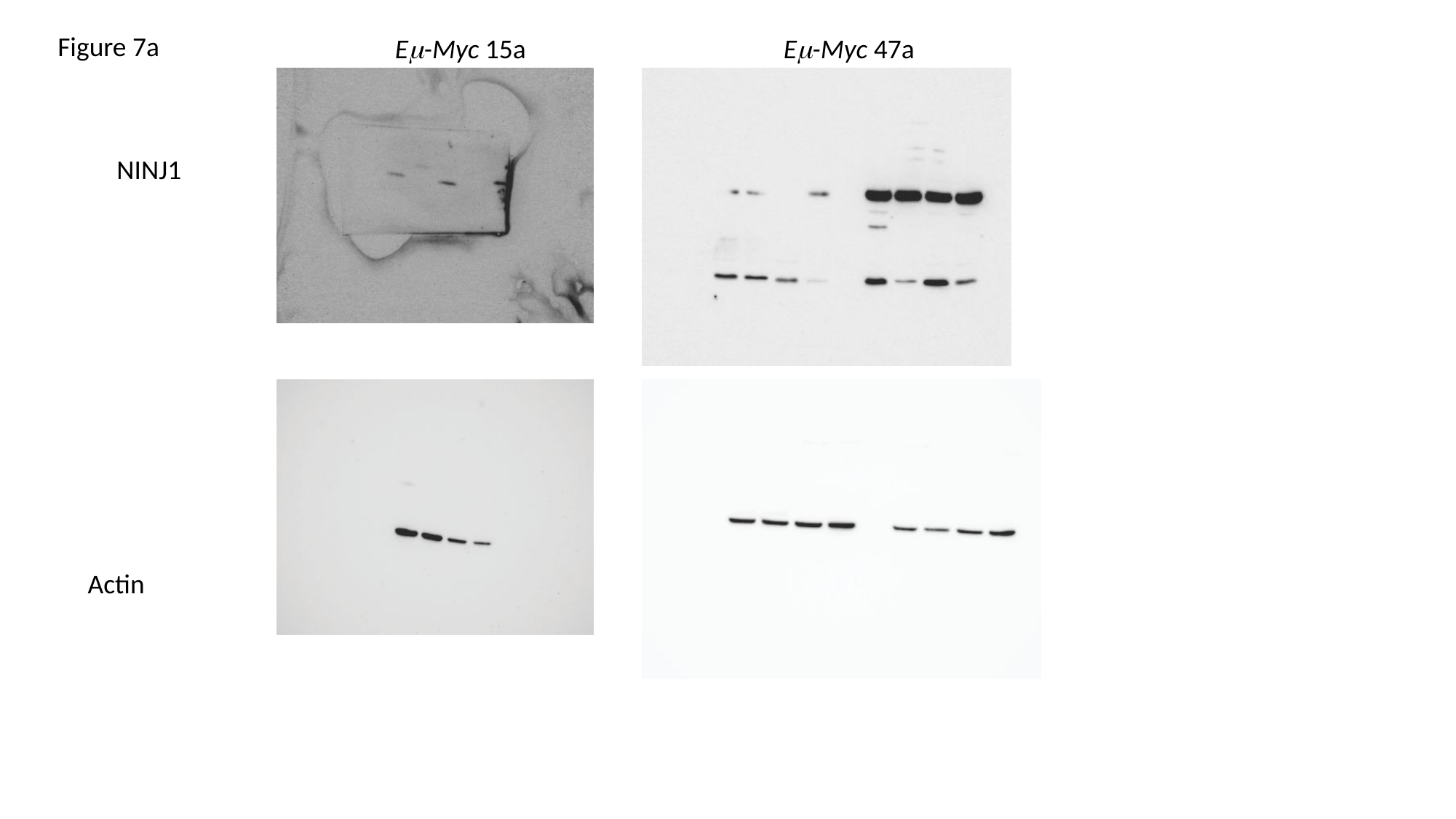

Figure 7a
Em-Myc 15a
Em-Myc 47a
NINJ1
Actin

## Slide 9
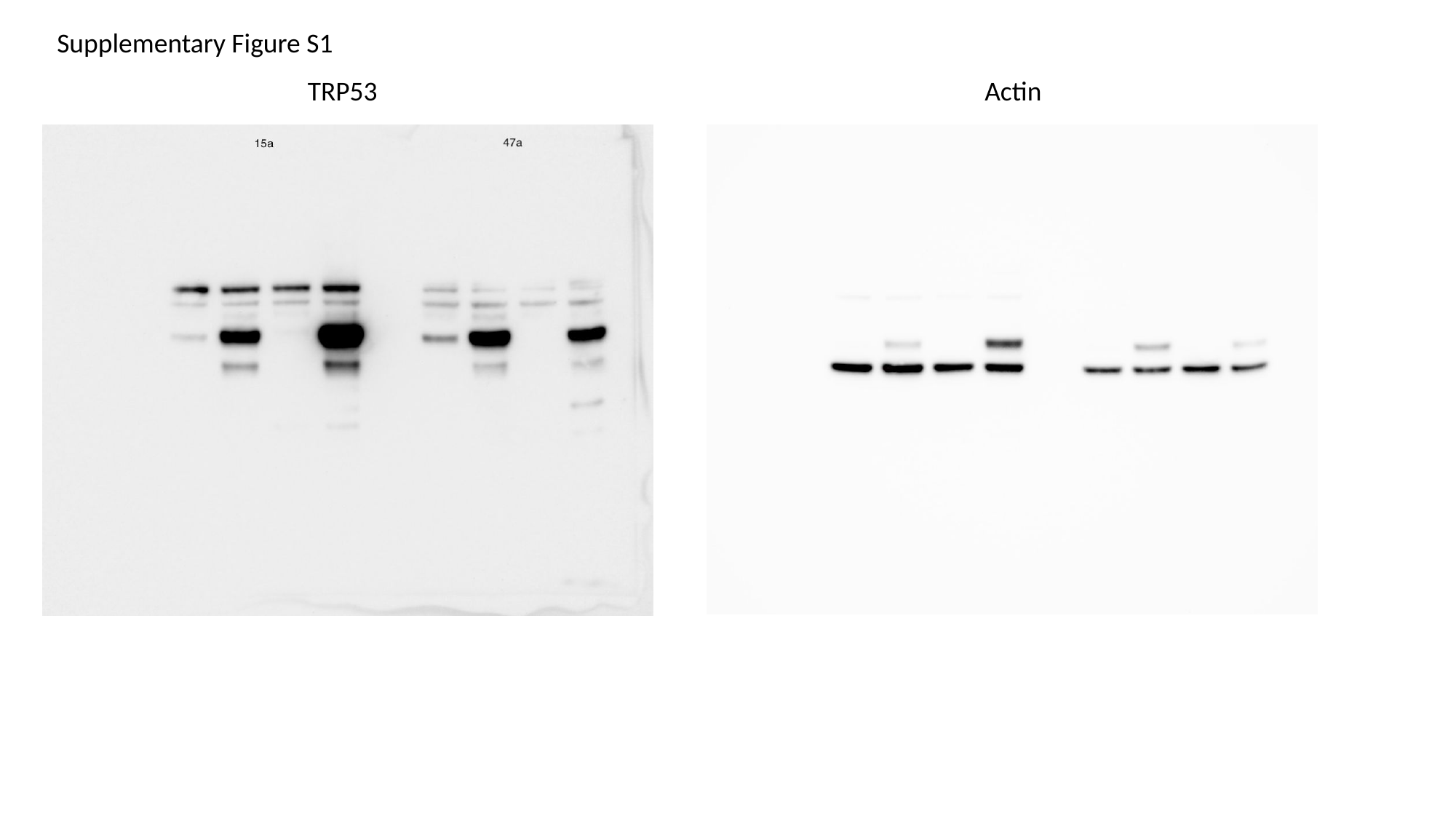

Supplementary Figure S1
TRP53
Actin

## Slide 10
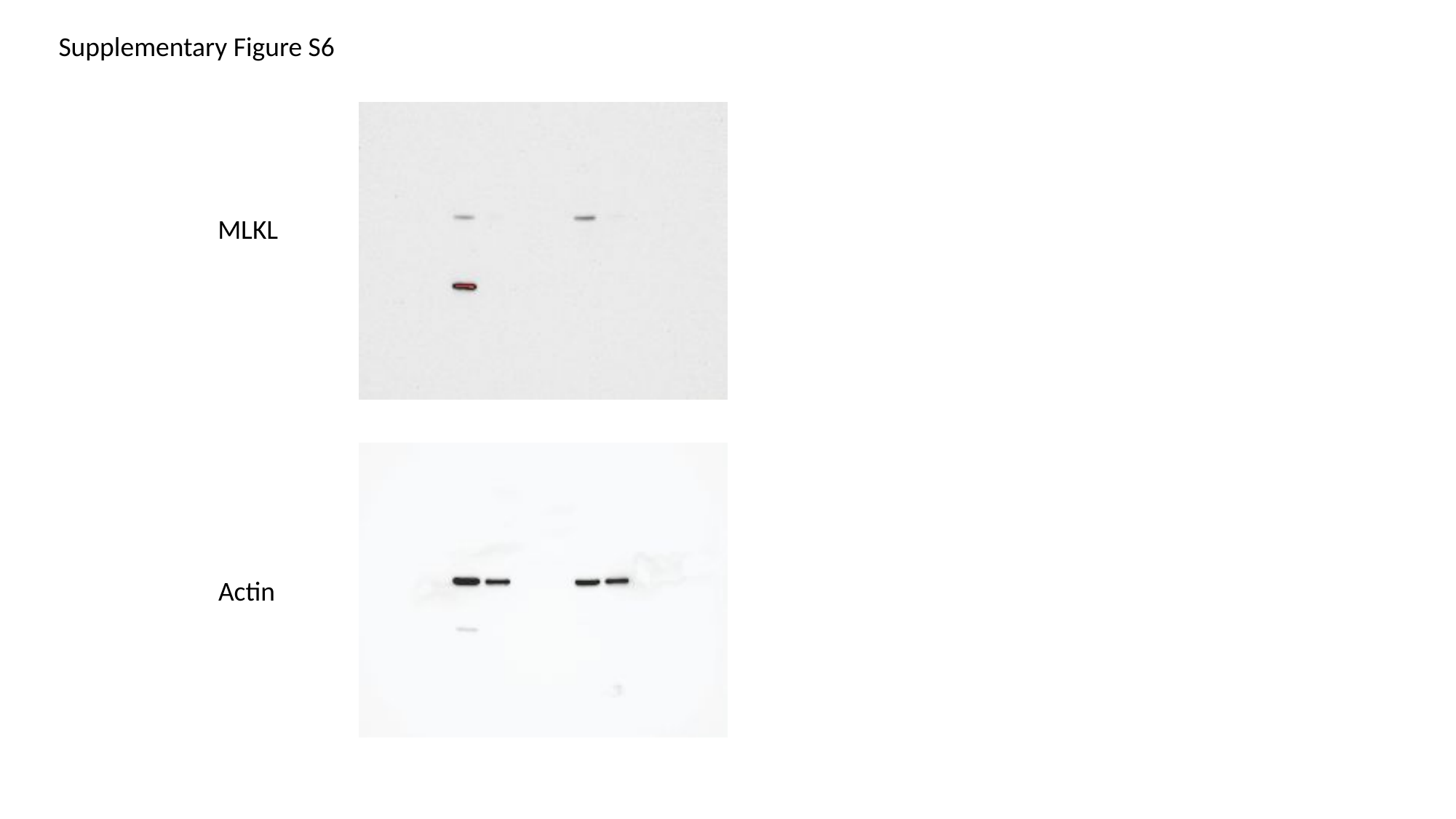

Supplementary Figure S6
MLKL
Actin
